# Supplementary material for: Cryo-EM reveals that iRhom2 restrains ADAM17 protease activity to control the release of growth factor and inflammatory signals
Source: Mol Cell. 2024 Jun 6;84(11):2152–2165.e5. doi: 10.1016/j.molcel.2024.04.025 (PMC11248996; doi:10.1016/j.molcel.2024.04.025)
Supplement: Document S1. Figures S1–S5 and Table S1 [file mmc1.pdf]

Molecular Cell, Volume *84*

Supplemental information

Cryo-EM reveals that iRhom2 restrains ADAM17 protease activity to control the release of growth factor and inflammatory signals

Fangfang Lu, Hongtu Zhao, Yaxin Dai, Yingdi Wang, Chia-Hsueh Lee, and Matthew Freeman

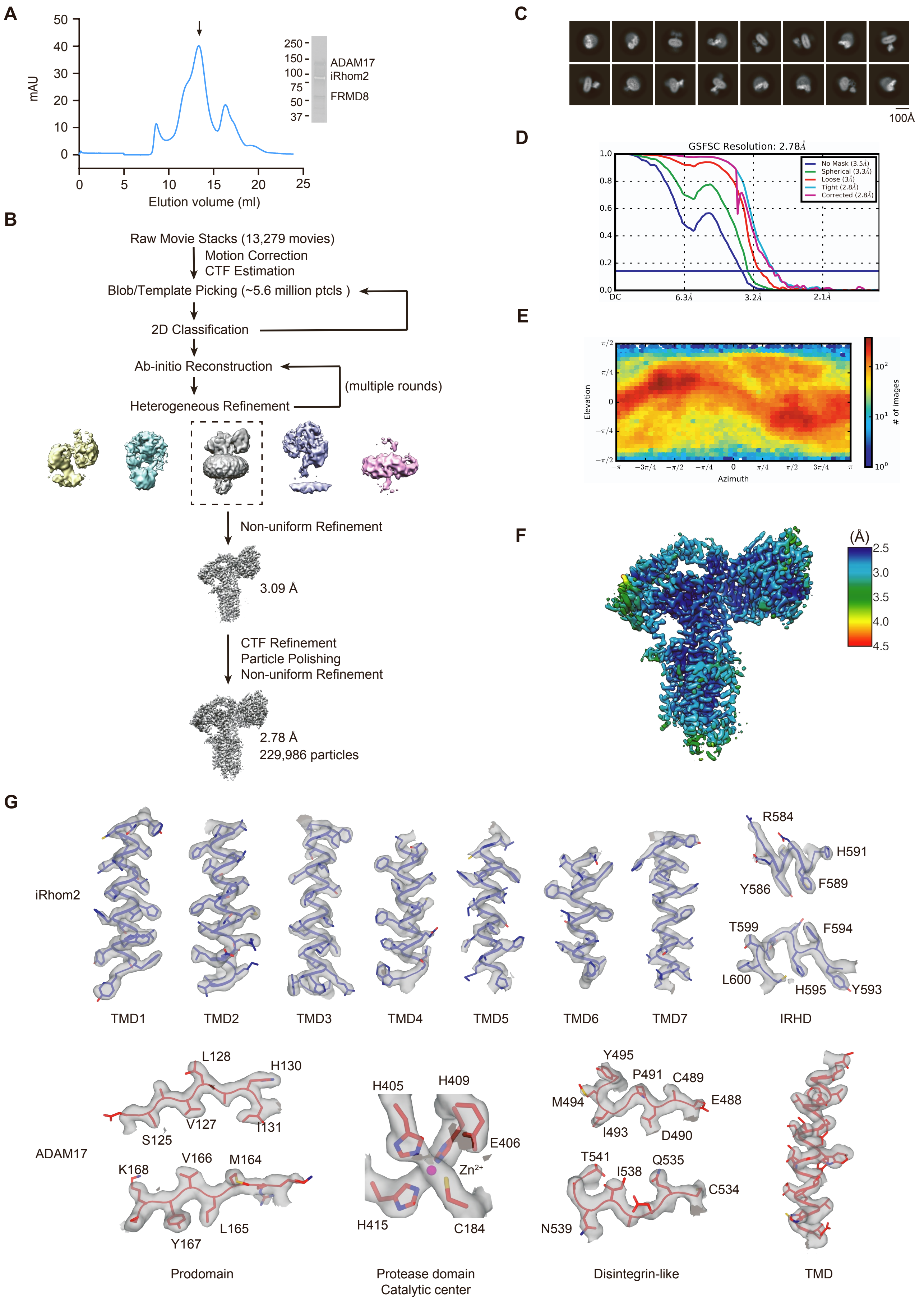
A
mAU
Elution volume (ml)
250
150
100
75
50
37
ADAM17
iRhom2
FRMD8
B
Raw Movie Stacks (13,279 movies)
Motion Correction
CTF Estimation
Blob/Template Picking (~5.6 million ptcls)
2D Classification
Ab-initio Reconstruction
(multiple rounds)
Heterogeneous Refinement
Non-uniform Refinement
3.09 Å
CTF Refinement
Particle Polishing
Non-uniform Refinement
2.78 Å
229,986 particles
C
100Å
D
GSFSC Resolution: 2.78Å
No Mask (3.5Å)
Spherical (3.3Å)
Loose (3Å)
Tight (2.8Å)
Corrected (2.8Å)
E
Elevation
Azimuth
# of images
F
(Å)
2.5
3.0
3.5
4.0
4.5
G
iRhom2
TMD1
TMD2
TMD3
TMD4
TMD5
TMD6
TMD7
R584
H591
Y586
F589
T599
F594
L600
H595
Y593
IRHD
ADAM17
L128
H130
V127
I131
S125
K168
V166
M164
L165
Y167
Prodomain
H405
H409
E406
Zn2+
H415
C184
Protease domain
Catalytic center
Y495
P491
C489
M494
E488
I493
D490
T541
I538
Q535
C534
N539
Disintegrin-like
TMD

Figure S1 Cryo-EM analyses of the human ADAM17/iRhom2 sheddase complex, related to Figure 1.

(A) Representative size exclusion chromatography profile and SDS-PAGE analysis of the purified ADAM17/iRhom2 complex. **(B)** Summary of image processing procedures of the ADAM17/iRhom2 complex. All procedures were done in cryoSPARC, except for particle polishing which was done in RELION. **(C)** Representative 2D class averages. **(D)** Fourier shell correlation (FSC) curves between two half maps. **(E)** Angular distribution of particles for the final 3D reconstructions. **(F)** Local resolution of the cryo-EM density. The map is colored according to local resolution, estimated using cryoSPARC. **(G)** cryo-EM densities of iRhom2 and ADAM17.

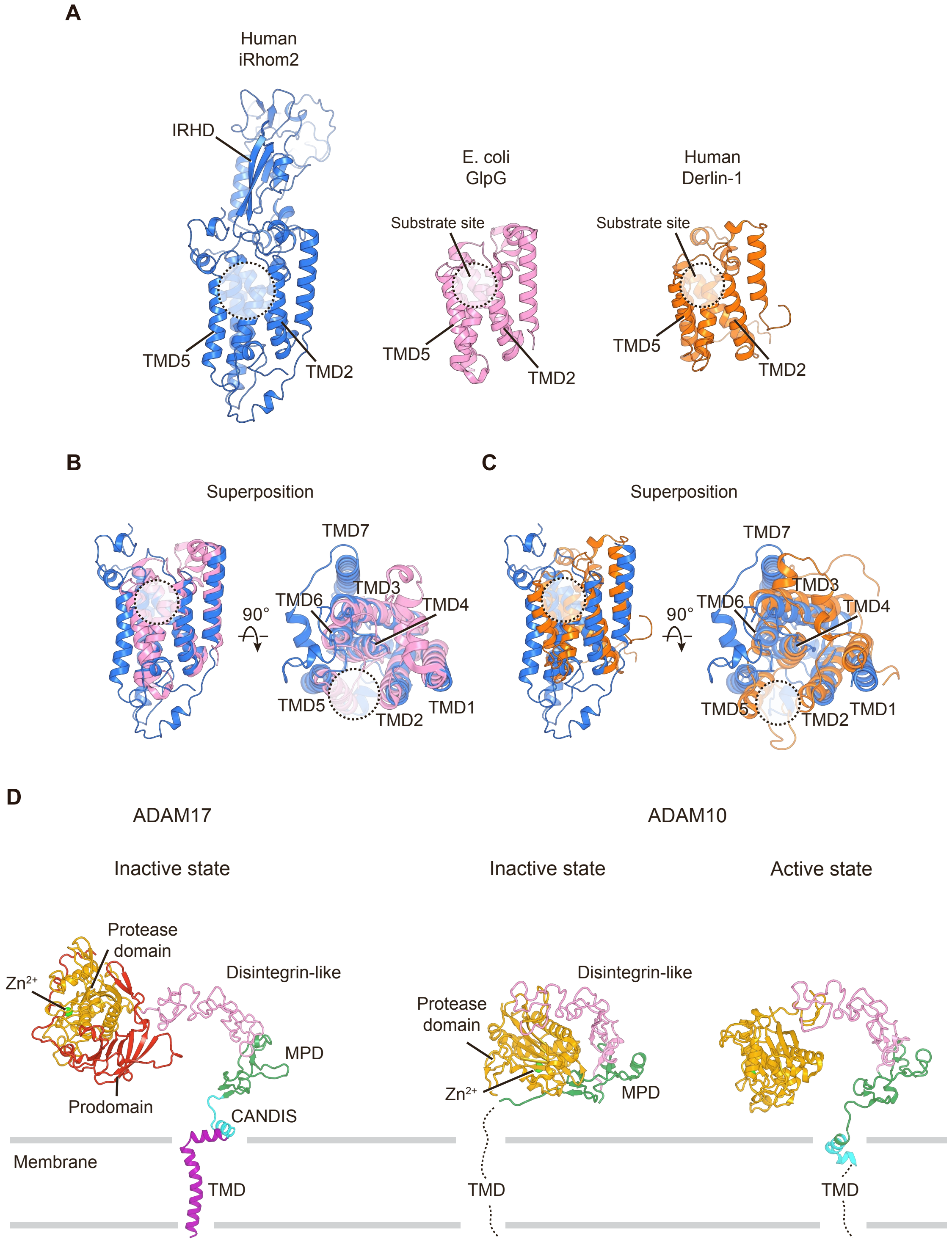

A
Human
iRhom2
IRHD
TMD5
TMD2
E. coli
GlpG
Substrate site
TMD5
TMD2
Human
Derlin-1
Substrate site
TMD5
TMD2
B
Superposition
90°
TMD7
TMD3
TMD6
TMD4
TMD5
TMD2
TMD1
C
Superposition
90°
TMD7
TMD3
TMD6
TMD4
TMD5
TMD2
TMD1
D
ADAM17
Inactive state
Protease
domain
Zn2+
Disintegrin-like
MPD
Prodomain
CANDIS
Membrane
TMD
ADAM10
Inactive state
Active state
Disintegrin-like
Protease
domain
Zn2+
MPD
TMD
TMD

Figure S2 Comparison of ADAM17/iRhom2 complex with related structures, related to Figures 1 and 2.

(A) Structures of human iRhom2, *E. coli* GlpG (PDB: 2IC8) and human derlin-1 (PDB: 7CZB). **(B)** Superposition between iRhom2 and GlpG. **(C)** Superposition between iRhom2 and derlin-1. **(D)** Structural comparison of ADAM17 and ADAM10 (PDB: 6BE6 and 8ESV). The three structures are aligned based on disintegrin-like domains. Note that ADAM10's protease domain interacts with the MPD in the inactive state. Such an interaction does not exist in the ADAM17 sheddase complex. The catalytic Zn^{2+} ions are shown as green spheres. The approximate membrane boundaries are represented by grey bars.

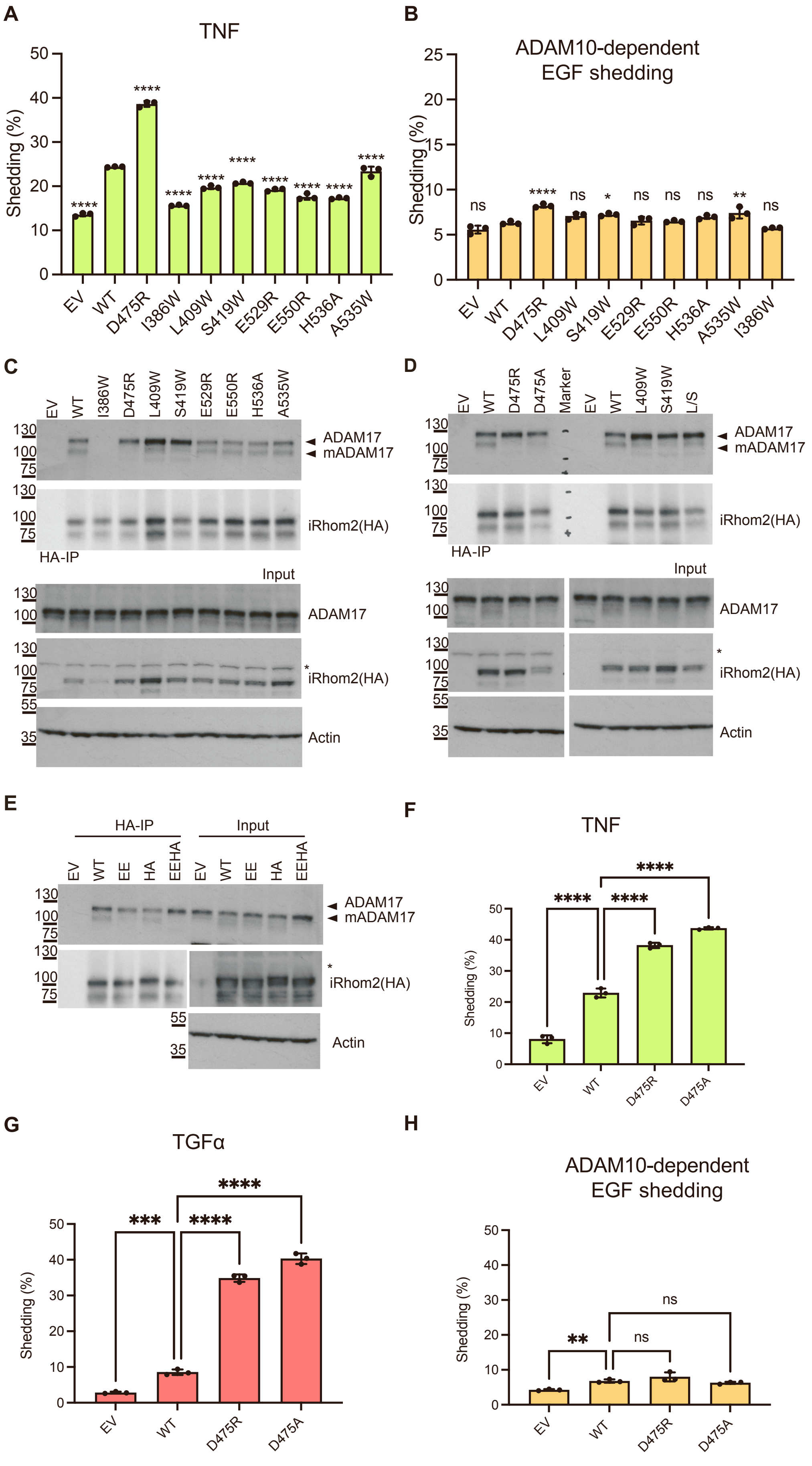

A
TNF
Shedding (%)
0
10
20
30
40
50
****
****
****
****
****
****
****
****
****
EV
WT
D475R
I386W
L409W
S419W
E529R
E550R
H536A
A535W
B
ADAM10-dependent
EGF shedding
Shedding (%)
0
5
10
15
20
25
ns
****
ns
*
ns
ns
ns
**
ns
EV
WT
D475R
L409W
S419W
E529R
E550R
H536A
A535W
I386W
C
EV
WT
I386W
D475R
L409W
S419W
E529R
E550R
H536A
A535W
130
100
75
ADAM17
mADAM17
iRhom2(HA)
HA-IP
Input
ADAM17
iRhom2(HA)
55
35
Actin
D
EV
WT
D475R
D475A
Marker
EV
WT
L409W
S419W
L/S
ADAM17
mADAM17
iRhom2(HA)
HA-IP
Input
ADAM17
iRhom2(HA)
Actin
E
HA-IP
Input
EV
WT
EE
HA
EEHA
ADAM17
mADAM17
iRhom2(HA)
Actin
F
TNF
Shedding (%)
****
****
****
EV
WT
D475R
D475A
G
TGFα
Shedding (%)
***
****
****
EV
WT
D475R
D475A
H
ADAM10-dependent
EGF shedding
Shedding (%)
**
ns
ns
EV
WT
D475R
D475A

Figure S3 Mutations at D475 in iRhom2 increases ADAM17-dependent growth factor and cytokine release, without affecting ADAM10 activity, related to Figures 3 and 4.

(A, B, F-H) iRhom1/2 DKO HEK cells were transfected with empty vector (EV) or different iRhom2 single point mutants together with AP-tagged ADAM17 substrates, tumor necrosis factor (TNF), transforming growth factor alpha (TGFα), or ADAM10 substrate epidermal growth factor (EGF). The growth medium was collected overnight and used for the AP-shedding assay. Error bars represent standard deviations (n=3, three transfectants). A Dunnett's test is performed by computing a Student's t-statistic for each transfection condition compared to the WT iRhom2 condition. ****=$p<0.0001$, ***=$p<0.001$, **=$p<0.01$, *=$p<0.05$, ns=not significant. Data are representative of three independent experiments. **(C-E)** iRhom1/2 DKO HEK cells were transfected with empty vector (EV) or different iRhom2 variants (L/S: L409W+ S419W, EE: E529R/E550R, HA: H536A/A535W, EEHA: E529R/E550R/H536A/A535W). HA-based immunoprecipitates and lysates were blotted for ADAM17, HA (iRhom2) and Actin. * indicates non-specific bands.

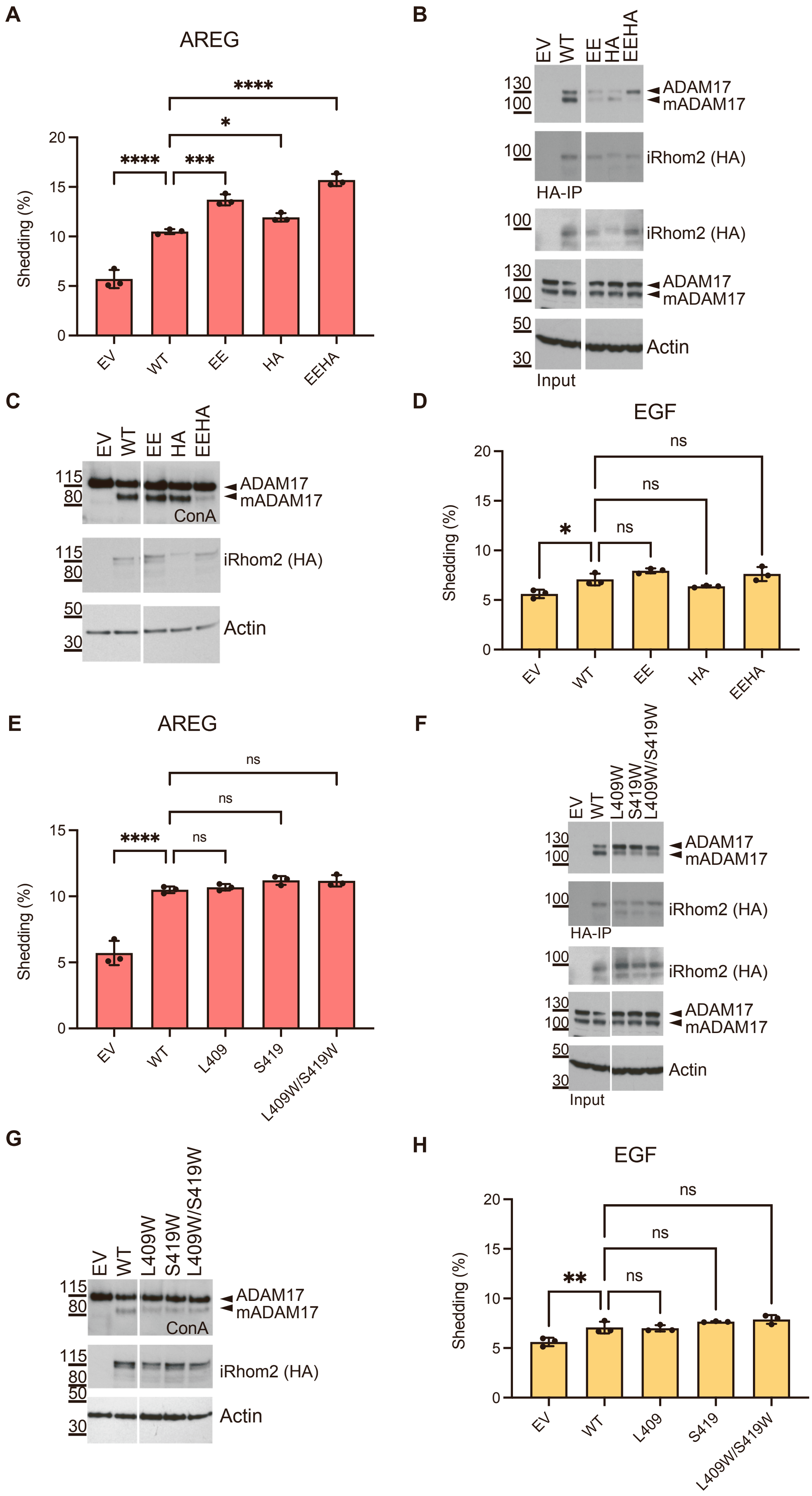
A
AREG
Shedding (%)
0
5
10
15
20
****
***
*
****
EV
WT
EE
HA
EEHA
B
EV
WT
EE
HA
EEHA
130
100
ADAM17
mADAM17
100
iRhom2 (HA)
HA-IP
100
iRhom2 (HA)
130
100
ADAM17
mADAM17
50
30
Actin
Input
C
EV
WT
EE
HA
EEHA
115
80
ADAM17
mADAM17
ConA
115
80
iRhom2 (HA)
50
30
Actin
D
EGF
Shedding (%)
0
5
10
15
20
*
ns
ns
ns
EV
WT
EE
HA
EEHA
E
AREG
Shedding (%)
0
5
10
15
****
ns
ns
ns
EV
WT
L409
S419
L409W/S419W
F
EV
WT
L409W
S419W
L409W/S419W
130
100
ADAM17
mADAM17
100
iRhom2 (HA)
HA-IP
100
iRhom2 (HA)
130
100
ADAM17
mADAM17
50
30
Actin
Input
G
EV
WT
L409W
S419W
L409W/S419W
115
80
ADAM17
mADAM17
ConA
115
80
iRhom2 (HA)
50
30
Actin
H
EGF
Shedding (%)
0
5
10
15
20
**
ns
ns
ns
EV
WT
L409
S419
L409W/S419W

Figure S4 Disrupting interaction at the Interfaces 2 and 4 had limited effects on ADAM17 activity, related to Figure 3.

(A) and **(D),** iRhom1/2 DKO HEK cells were transfected with empty vector (EV) or different iRhom2 Interface 2 mutants (EE: E529R/E550R, HA: H536A/A535W, EEHA: E529R/E550R/H536A/A535W) together with AP-tagged AREG as ADAM17 substrate or AP-tagged EGF as ADAM10 substrate in (D). Error bars represent standard deviations (n=3，three transfectants). **(B)** iRhom1/2 DKO HEK cells were transfected with WT or iRhom2 Interface 2 mutants (EE, HA, EEHA). HA-based immunoprecipitates and lysates were blotted with endogenous ADAM17, HA (iRhom2), and actin. **(C)** Concanavalin A (ConA) enrichment was performed to the lysates from the shedding assay in (A). **(E)** and **(H),** iRhom1/2 DKO HEK cells were transfected with empty vector (EV) or different iRhom2 Interface 4 mutants (L409W, S419W, L/S: L409W+ S419W) together with AP-tagged AREG as ADAM17 substrate or AP-tagged EGF as ADAM10 substrate in (H). Error bars represent standard deviations (n=3, three transfectants). **(F)** iRhom1/2 DKO HEK cells were transfected with WT or iRhom2 Interface 4 mutants (L409W, S419W, L/S). HA-based immunoprecipitates and lysates were blotted with endogenous ADAM17, HA (iRhom2), and actin. **(G)** Concanavalin A (ConA) enrichment was performed to the lysates from the shedding assay in (E). Data are representative of three independent experiments (A-H). For figures A, D, E, H, a Dunnett's test is performed by computing a Student's t-statistic for each transfection condition compared to the WT iRhom2 condition. ****=$p<0.0001$, ***=$p<0.001$, **=$p<0.01$, *=$p<0.05$, ns= not significant. Note: EV, WT conditions are on the same immunoblot as the different mutant conditions, with superfluous lanes removed to make comparison easier. (B) and (F) show different mutants from one HA-IP experiment and therefore share the same control conditions.

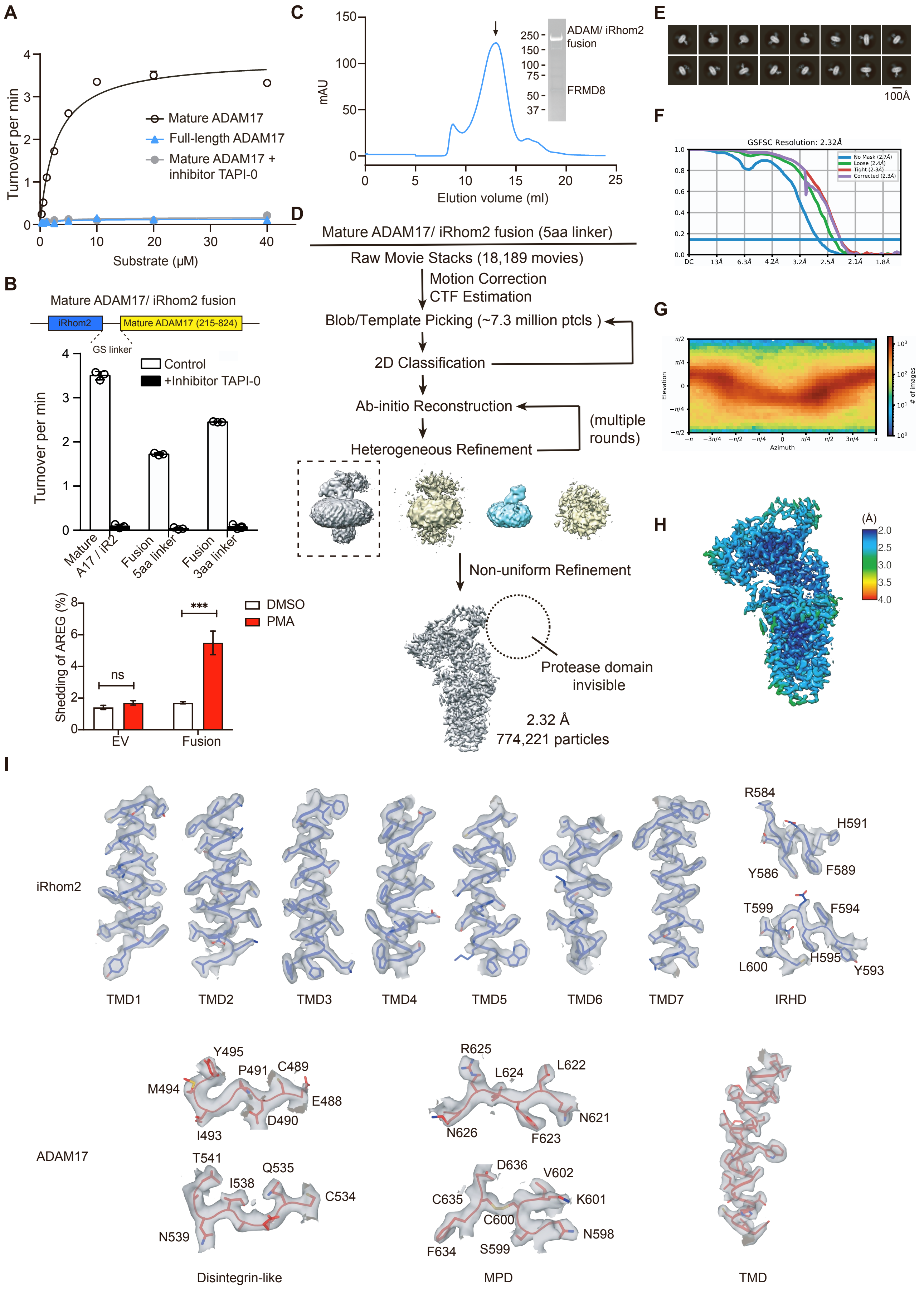

A
Turnover per min
Substrate (μM)
Mature ADAM17
Full-length ADAM17
Mature ADAM17 + inhibitor TAPI-0
B
Mature ADAM17/ iRhom2 fusion
iRhom2
Mature ADAM17 (215-824)
GS linker
Control
+Inhibitor TAPI-0
Turnover per min
Mature A17 / iR2
Fusion 5aa linker
Fusion 3aa linker
Shedding of AREG (%)
DMSO
PMA
ns
***
EV
Fusion
C
mAU
Elution volume (ml)
ADAM/ iRhom2 fusion
FRMD8
D
Mature ADAM17/ iRhom2 fusion (5aa linker)
Raw Movie Stacks (18,189 movies)
Motion Correction
CTF Estimation
Blob/Template Picking (~7.3 million ptcls)
2D Classification
Ab-initio Reconstruction
Heterogeneous Refinement
(multiple rounds)
Non-uniform Refinement
Protease domain invisible
2.32 Å
774,221 particles
E
100Å
F
GSFSC Resolution: 2.32Å
No Mask (2.7Å)
Loose (2.4Å)
Tight (2.3Å)
Corrected (2.3Å)
G
Elevation
Azimuth
# of images
H
(Å)
I
iRhom2
TMD1
TMD2
TMD3
TMD4
TMD5
TMD6
TMD7
IRHD
R584
H591
Y586
F589
T599
F594
L600
H595
Y593
ADAM17
Y495
C489
P491
M494
E488
D490
I493
T541
Q535
I538
C534
N539
Disintegrin-like
R625
L624
L622
N621
N626
F623
D636
V602
C635
K601
C600
N598
F634
S599
MPD
TMD

Figure S5 Functional and cryo-EM analyses of mature ADAM17/iRhom2 complexes, related to Figure 5.

(A) Protease activity of the purified mature ADAM17/iRhom2 complex and the full length. **(B)** Upper panel: Protease activity of the purified, mature ADAM17/iRhom2 fusions. Lower panel: AP-shedding assay of iRhom1/2 DKO HEK cells which were transfected with empty vector (EV) or an iRhom2-matureADAM17 fusion construct (Fusion) together with the ADAM17 substrate alkaline phosphatase (AP)-tagged AREG. A Student's t-statistic test was performed. ***=p<0.001, ns= not significant; n=3, three transfectants. In (A) and (B), error bars represent standard deviations. **(C)** Representative size exclusion chromatography profile and SDS-PAGE analysis of the mature ADAM17/iRhom2 fusion (5 aa linker construct). **(D)** Summary of image processing procedures of the mature ADAM17/iRhom2 fusion (5 aa linker construct). All procedures were done in cryoSPARC, except for particle polishing which was done in RELION. **(E)** Representative 2D class averages. **(F)** Fourier shell correlation (FSC) curves between two half maps. **(G)** Angular distribution of particles for the final 3D reconstructions. **(H)** Local resolution of the cryo-EM density. The map is colored according to local resolution, estimated using cryoSPARC. **(I)** cryo-EM densities of iRhom2 and mature ADAM17.

Table S1. Table of mutants, related to Figure 3.

Interface	iRhom2 domain	iRhom2 isoform2	Potential ADAM17 interacting residues	ADAM17 domain
4	IRHD	L409W<br>S419W	V120<br>H118	Prodomain
3	IRHD	D475R	S63 or T64 or T62	Prodomain
2	IRHD	E529R<br>E550R<br>H536A<br>A535W	K626<br>R625<br>K628<br>S590	Membrane proximal domain (MPD)
1	TMD1	I386W	V673, V676, L677	Transmembrane domain (TMD)
Rationale of mutagenesis design	To ensure disruption of the interaction interface, the changes of amino acids were designed to be profound. All small residues (L, S, A, I, V) were mutated to a bulky residue W. For charged residues, the charge was reversed. For instance, an E was mutated into an R or vice versa. In addition, H was mutated to A.			
