# Supplementary material for: Cryo-EM reveals that iRhom2 restrains ADAM17 protease activity to control the release of growth factor and inflammatory signals
Source: Mol Cell. 2024 Jun 6;84(11):2152–2165.e5. doi: 10.1016/j.molcel.2024.04.025 (PMC11248996; doi:10.1016/j.molcel.2024.04.025)
Supplement: Document S2. Article plus supplemental information [file mmc2.pdf]

Molecular Cell

Article

Cryo-EM reveals that iRhom2 restrains ADAM17 protease activity to control the release of growth factor and inflammatory signals

Graphical abstract

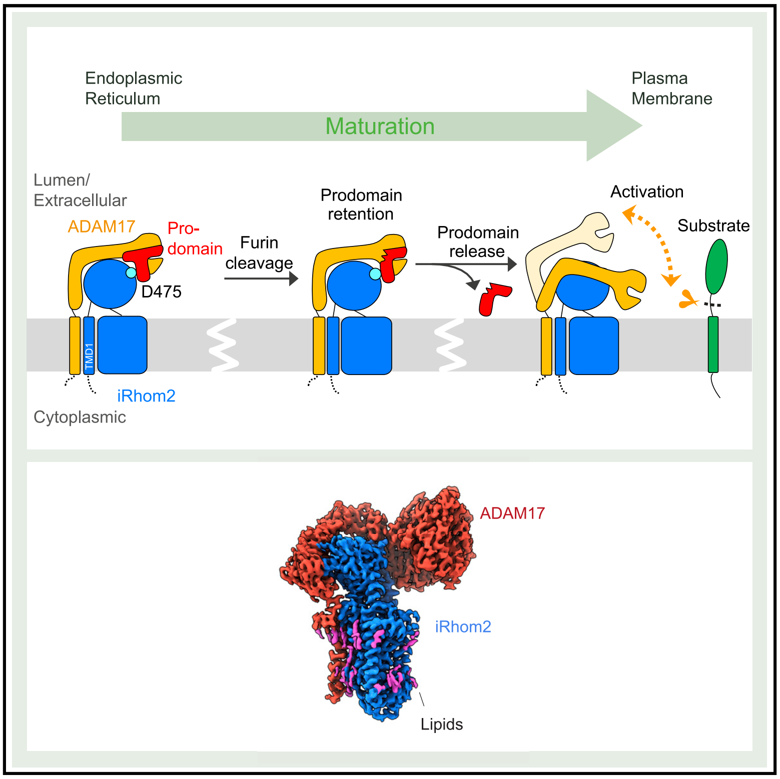

Authors

Fangfang Lu, Hongtu Zhao, Yaxin Dai, Yingdi Wang, Chia-Hsueh Lee, Matthew Freeman

Correspondence

zhao_hongtu@gzlab.ac.cn (H.Z.),
chiahsueh.lee@stjude.org (C.-H.L.),
matthew.freeman@path.ox.ac.uk (M.F.)

In brief

The cryo-EM structures of human ADAM17/iRhom2 sheddase complex identify the interaction interfaces responsible for controlling ADAM17 trafficking, maturation, and activity. Functional studies reveal how the pseudoprotease iRhom2 has evolved to regulate the potent signaling enzyme ADAM17, ensuring fidelity of growth factor and inflammatory signaling.

Highlights

- Human ADAM17/iRhom2 sheddase complex structures determined by cryo-EM
- Transmembrane domain interaction is essential for complex assembly and ADAM17 maturation
- iRhom2 controls substrate shedding by regulating release of the ADAM17 prodomain
- Mature ADAM17 is highly dynamic, contributing to its versatility as a sheddase

Lu et al., 2024, Molecular Cell *84*, 2152–2165
June 6, 2024 © 2024 The Authors. Published by Elsevier Inc.
https://doi.org/10.1016/j.molcel.2024.04.025

CellPress
OPEN ACCESS

Molecular Cell

Article

Cryo-EM reveals that iRhom2 restrains ADAM17 protease activity to control the release of growth factor and inflammatory signals

Fangfang Lu,[1,3] Hongtu Zhao,[2,3,4,*] Yaxin Dai,[2] Yingdi Wang,[2] Chia-Hsueh Lee,[2,*] and Matthew Freeman[1,5,*]
[1]Sir William Dunn School of Pathology, University of Oxford, South Parks Road, Oxford OX1 3RE, UK
[2]Department of Structural Biology, St. Jude Children's Research Hospital, Memphis, TN 38105, USA
[3]These authors contributed equally
[4]Present address: Guangzhou National Laboratory, Guangzhou, Guangdong Province 510005, China
[5]Lead contact
*Correspondence: zhao_hongtu@gzlab.ac.cn (H.Z.), chiahsueh.lee@stjude.org (C.-H.L.), matthew.freeman@path.ox.ac.uk (M.F.)
https://doi.org/10.1016/j.molcel.2024.04.025

SUMMARY

A disintegrin and metalloprotease 17 (ADAM17) is a membrane-tethered protease that triggers multiple signaling pathways. It releases active forms of the primary inflammatory cytokine tumor necrosis factor (TNF) and cancer-implicated epidermal growth factor (EGF) family growth factors. iRhom2, a rhomboid-like, membrane-embedded pseudoprotease, is an essential cofactor of ADAM17. Here, we present cryoelectron microscopy (cryo-EM) structures of the human ADAM17/iRhom2 complex in both inactive and active states. These reveal three regulatory mechanisms. First, exploiting the rhomboid-like hallmark of TMD recognition, iRhom2 interacts with the ADAM17 TMD to promote ADAM17 trafficking and enzyme maturation. Second, a unique iRhom2 extracellular domain unexpectedly retains the cleaved ADAM17 inhibitory prodomain, safeguarding against premature activation and dysregulated proteolysis. Finally, loss of the prodomain from the complex mobilizes the ADAM17 protease domain, contributing to its ability to engage substrates. Our results reveal how a rhomboid-like pseudoprotease has been repurposed during evolution to regulate a potent membrane-tethered enzyme, ADAM17, ensuring the fidelity of inflammatory and growth factor signaling.

INTRODUCTION

Proteolytic release, or shedding, of membrane-tethered ligands is a primary trigger of signaling between cells[1,2] and has been linked to the pathogenesis of multiple inflammatory and other diseases.[3] A major player in this process is the cell surface metalloprotease ADAM17 (a disintegrin and metalloprotease 17), which has a central role in regulating inflammation and growth factor signaling.[4] ADAM17 is responsible for the cleavage and release of more than eighty different substrates, including the primary inflammatory cytokine tumor necrosis factor (TNF) and growth factors of the epidermal growth factor (EGF) family.[5] The consequent biological and medical importance of ADAM17, combined with the proven clinical importance of TNF blockade, has led to extensive efforts to develop specific inhibitors that can block ADAM17 activity.[6] Nevertheless, our mechanistic and structural understanding of this protease is limited.[4,7,8] ADAM17 is a single-pass transmembrane protease.[9,10] In addition to its extracellular protease domain, it features an N-terminal inhibitory prodomain, a disintegrin-like domain, a membrane proximal domain (MPD), a conserved stalk region (CANDIS), a transmembrane domain (TMD), and a cytoplasmic domain.[6,11] All of these domains are known to modulate ADAM17's activity,[8,12–14] but their spatial organization and regulatory mechanisms have been poorly understood. The N-terminal prodomain appears to be particularly important: at its C terminus there is a cleavage site for the proconvertase enzyme furin, and processing at this site is proposed to be essential for the release of the inhibitory prodomain and subsequent activation of ADAM17.[15]

Overall, the mechanisms underlying the precise regulation of ADAM17 sheddase activity have remained elusive, although it has recently become apparent that iRhoms are important components of the process. iRhoms are highly specific ADAM17 regulatory cofactors with corresponding functions in inflammation, immune and growth factor signaling, and cancer.[16–22] They belong to the rhomboid-like superfamily, which comprises both the rhomboid intramembrane proteases and the more recently discovered pseudoproteases.[23] The superfamily has a wide range of known functions, including, but not limited to, signaling, mitochondrial morphology, parasitic host cell invasion, and endoplasmic reticulum (ER)-associated protein degradation (ERAD).[24] The defining feature of all rhomboid-like proteins is a conserved domain of at least six TMDs, which has been proposed to mediate specific TMD recognition of substrates or

 © 2024 The Authors. Published by Elsevier Inc.
This is an open access article under the CC BY license (http://creativecommons.org/licenses/by/4.0/).

CellPress
OPEN ACCESS

client proteins.[24] Although rhomboid proteases have been thoroughly studied,[25–28] our understanding of rhomboid pseudoproteases like iRhoms remains very limited. All iRhoms have a highly conserved and unique extracellular domain between TMD1 and TMD2, the iRhom homology domain (IRHD).[24] The IRHD is indispensable to the function of the sheddase complex,[18,29] but the underlying mechanism is unknown.

Genetic and cellular studies have revealed at least two distinct roles for iRhoms in ADAM17 regulation. Maturation of ADAM17 occurs as the protein is trafficked through the Golgi apparatus, where furin cleaves the peptide bond between the prodomain and the rest of the protein. The first role of iRhoms involves this trafficking and maturation process: in the absence of iRhoms, ADAM17 cannot leave the ER, thus preventing its subsequent maturation and its transport to the plasma membrane.[16,17] The second role of iRhoms occurs post furin maturation: they remain associated with ADAM17 when the complex is trafficked to the cell surface. In response to intracellular signals, the iRhom cytoplasmic tail is phosphorylated, which induces the binding of 14-3-3 proteins to the complex, a poorly defined weakening of the ADAM17-iRhom2 interaction, and the eventual activation of ADAM17.[18,19] Additionally, iRhoms may contribute to ADAM17 substrate selectivity.[30] However, the molecular mechanisms by which iRhoms control these different processes are unclear. This gap in our knowledge hinders the development of therapeutics targeting aberrant cytokine and growth factor release mediated by the ADAM17/iRhom complex.

Here, we report structures of the human ADAM17/iRhom2 sheddase complex in both inactive and active states. These provide structural insights into both full-length ADAM17 and iRhoms and reveal the architecture of the sheddase complex. They also uncover the distinct interfaces between iRhom2 and ADAM17 that participate in regulating ADAM17 activity. These include an unexpected interaction between the iRhom2 IRHD and the ADAM17 prodomain, which is maintained even after furin cleaves the peptide bond between the prodomain and the active enzyme. Notably, once the prodomain does leave the complex, not only is the catalytic site available but the protease also becomes more flexible, implying that the prodomain restrains conformational flexibility of the enzyme in addition to competitively inhibiting the active site. Overall, our structures of the human ADAM17/iRhom2 complex reveal the essential regulatory mechanisms that iRhom2 employs to regulate ADAM17-mediated inflammation and growth factor signaling.

RESULTS

Structure determination of the ADAM17/iRhom2 complex

To study the human sheddase complex, we coexpressed full-length human ADAM17 and iRhom2 in mammalian human embryonic kidney (HEK) cells.[9,10] We focused on iRhom2 because it has been more extensively characterized than the only other human paralog, iRhom1.[31] We also coexpressed FRMD8 (FERM domain-containing protein 8), a recently discovered cytosolic protein that binds iRhom2. FRMD8 is dispensable for the formation of the complex, but it stabilizes the complex at the cell surface.[20,21] The coexpression of FRMD8 increased the protein yield of the sheddase complex for structural studies. After purification, we determined the structure of the complex at 2.8 Å resolution using single-particle cryoelectron microscopy (cryo-EM) (Figures 1 and S1). Although the density for FRMD8 and the intracellular regions of the complex is unresolved, the density for the majority of iRhom2 and ADAM17 is well defined (Figure S1). The density map is of high quality, allowing us to unambiguously build the structure of the entire extracellular and TMDs of the complex (Figure 1B).

Overall architecture and the structure of iRhom2

The sheddase complex consists of one ADAM17 and one iRhom2 molecule (Figure 1B). The two proteins interact in both extracellular and transmembrane regions. ADAM17 is shaped like an inverted L, with a globular head that extends outward and hangs over the large extracellular IRHD (Figures 1B and 1F). The IRHD protrudes about 45 Å above the membrane, acting as a buttress for ADAM17. It is stapled by 8 disulfide bonds and contains both α helices and β sheets (Figure 1C). The structure of the IRHD appears to be unprecedented, as no similar structures could be found in the Protein Data Bank or in the AlphaFold-predicted structure database of the human proteome.[32,33] The rest of iRhom2 adopts a fold reminiscent of rhomboid proteases (Figures S2A and S2B),[25,34] albeit with noticeable differences. For example, iRhom2 possesses an additional helix TMD7 (Figures 1C, 1D, and S2B), compared with the 6 TMDs in the available structures of bacterial rhomboid proteases.[27,34] Moreover, residues N-terminal to TMD1 form short helical stretches and a reentrant loop that inserts itself between TMD2 and TMD5 (Figures 1C and 1D). In rhomboid proteases and Derlin rhomboid pseudoproteases, TMD2 and TMD5 create a pathway for substrate entry and a docking site for other proteins (Figures S2A–S2C).[25,34–36] In contrast to these other members of the rhomboid-like superfamily,[25,37] iRhom2 employs a distinct interface to engage with ADAM17, depending on TMD1 (Figure 1E).

Structure of ADAM17 and basis of its prodomain inhibition

In our structure of the sheddase complex, the extracellular domain of ADAM17 adopts a fully extended conformation (Figure 2A). This contrasts sharply with the compact, closed, inactive conformation or the more open, active conformation observed for its closest homolog, ADAM10 (Figure S2D),[38,39] which does not interact with iRhom2.[19] ADAM17 is in an inactive state, with its uncleaved prodomain associated with the protease domain (Figure 2A). The ADAM17 prodomain forms a β-barrel core that binds to the protease domain. In addition, extended loops and β-sheets of the prodomain further encapsulate the protease domain. Similar to prodomains in other metalloproteinases, the ADAM17 prodomain contains a conserved cysteine-switch motif, where a cysteine residue is believed to block the active site.[40] Indeed, Cys184 coordinates the catalytic zinc ion along with His405, His409, and His419 from the protease domain, thereby shielding the core catalytic region (Figure 2B). However, prodomain mutants lacking this cysteine can still impede ADAM17 activity,[41,42] indicating additional mechanisms for prodomain inhibition. We find that the prodomain's

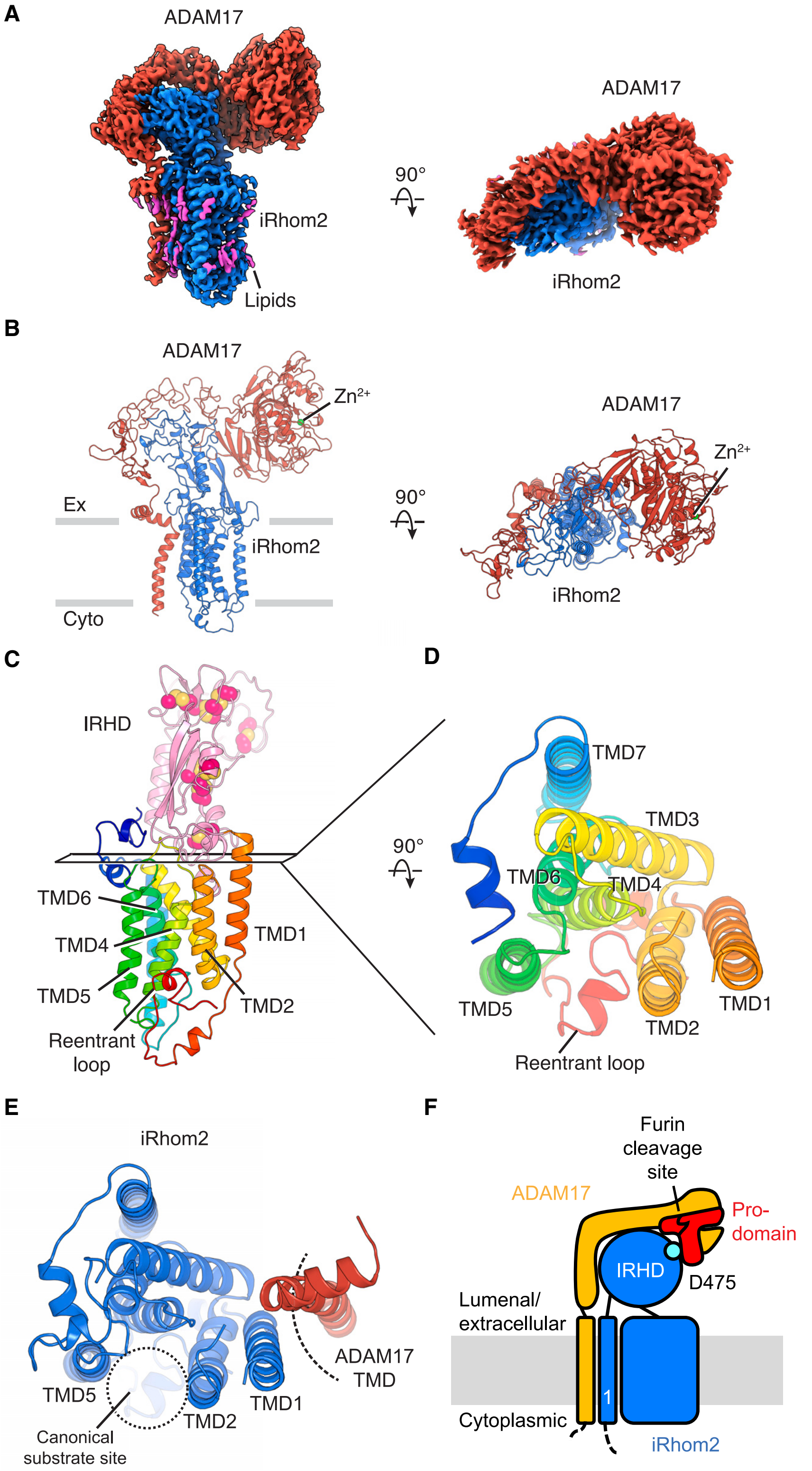

Figure 1. Architecture of the human ADAM17/iRhom2 sheddase complex

(A) Cryo-EM density map of the human ADAM17/iRhom2 sheddase complex, viewed from the side of the membrane (left) or from the extracellular space (right).

(B) Overall structure of the human ADAM17/iRhom2 complex. The catalytic Zn^{2+} is shown as a green sphere. The approximate membrane boundaries are represented by gray bars.

(C) Structure of iRhom2. Disulfide bonds in the IRHD (iRhom homology domain) are shown as spheres.

(D) Transmembrane domains of iRhom2, viewed from the extracellular side.

(E) Intramembrane interface between iRhom2 and ADAM17.

(F) A diagram of the sheddase complex. ADAM17 is shown in yellow, with its prodomain in red. iRhom2 is colored blue. The first transmembrane domain of iRhom2 is labeled "1." The key D475 residue (discussed below) is shown as a turquoise circle. The furin cleavage site is indicated.

See also Figures S1 and S2.

C-terminal loop occupies the entire substrate binding groove, effectively blocking substrate access to the catalytic site (Figure 2B). The short sequence segment around the furin cleavage site remains unresolved in the density map, indicating its flexibility, which may facilitate access to furin.

A feature of the inactive sheddase complex is that the prodomain interacts not only with the protease domain but also with the disintegrin-like domain and, unexpectedly, with the IRHD of iRhom2 (Figure 2C). The latter interaction raises the possibility that iRhom2 may directly regulate the release of the prodomain to modulate ADAM17 activation.

Dissecting the interactions between iRhom2 and ADAM17

To understand how iRhom2 regulates ADAM17, we focused on the four interfaces identified from the structure (Figure 3A). Interface 1 occurs within the TMDs, whereas interfaces 2–4 are between the IRHD of iRhom2 and the extracellular region of ADAM17. Intriguingly, two of these non-membrane interfaces (interfaces 3 and 4) involve the ADAM17 prodomain (Figure 3A). To investigate the functional significance of these interfaces, we performed systematic mutagenesis of relevant residues of iRhom2 to disrupt the observed interactions and expressed the mutant forms in HEK cells in which both iRhom1 and iRhom2 had been knocked out (double knockout [DKO] cells). Using a well-established assay to measure the unstimulated release of the ADAM17 substrate amphiregulin (AREG) tagged with alkaline phosphatase (AP),[18,20,22] we determined the effect of these

CellPress
OPEN ACCESS

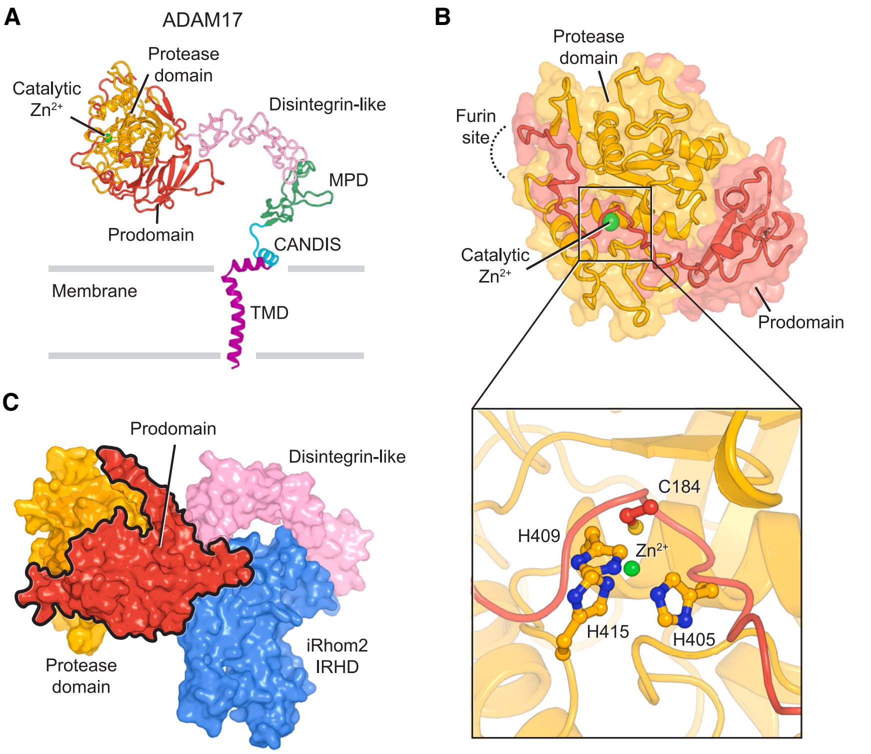

Figure 2. Structure of ADAM17 and the mechanism of prodomain inhibition

(A) Domain arrangement of ADAM17 in the sheddase complex. The catalytic Zn^{2+} is shown as a green sphere. The approximate membrane boundaries are represented by gray bars. MPD, membrane proximal domain. CANDIS, conserved ADAM17 dynamic interaction sequence.

(B) Structure of ADAM17 prodomain and protease domain. The furin site, not resolved in the structure, is indicated by a dashed line. The inset shows a zoomed-in view of the catalytic center of the protease domain.

(C) Interfaces between the prodomain and the rest of the complex. The extracellular domain of the ADAM17/iRhom2 complex is shown in surface representation.

See also Figure S2.

iRhom2 mutations (which are localized indistinguishably from wild-type [WT] iRhom2; not shown) on basal ADAM17 shedding activity (Figure 3B). As a control for the specificity of iRhom2 mutations, we examined their effects on the shedding activity of ADAM10, a related metalloprotease not regulated by iRhom2.[18,19] In parallel, we measured the ability of iRhom2 mutants to support ADAM17 maturation (Figure 3C) and assessed the physical interaction between the two proteins by co-immunoprecipitation (Figures 3D–3F). We note that the co-immunoprecipitation assay was performed in WT HEK cells in which endogenous iRhoms are present, thereby ensuring that ADAM17 maturation could occur, regardless of whether the mutant iRhom2 itself supports maturation. Similar immunoprecipitation results were also obtained using the iRhom1/2 DKO cells (Figures S3C–S3E).

The strongest effect was caused by disrupting interface 1, between TMD1 of iRhom2 and the single TMD of ADAM17 (Figure 3A). The I386W mutation in iRhom2 led to loss of ADAM17-dependent shedding of AREG (Figure 3B), loss of detectable mature ADAM17 (Figure 3C), and abolished physical interaction between iRhom2 and ADAM17 (Figure 3D). This effect was also seen with another ADAM17 substrate, TNF, the primary inflammatory cytokine (Figure S3A). As expected, ADAM10-dependent shedding activity was unaffected by iRhom2 I386W (Figure S3B). These results suggest that, without the TMD interaction between iRhom2 and ADAM17, ADAM17 does not leave the ER, thereby failing to mature or reach the plasma membrane from where substrates are normally shed. In line with this conclusion, a similar mutation in TMD1 of mouse iRhom2 was reported to abolish ADAM17-dependent TNF release.[43,44] Additionally, a TMD-swapping experiment further demonstrated the essential role of ADAM17's TMD in its regulation by iRhom.[45] The prominence of the TMD interaction within the sheddase complex strongly supports the idea that specific transmembrane recognition is a fundamental feature of the rhomboid-like structure.

Disruption of iRhom2/ADAM17 interfaces 2 and 4 also affected the complex, but in subtle ways. Single mutations at interface 2 (E529R, E550R, H536A, and A535W) had little effect on ADAM17 shedding of AREG and TNF, or its maturation, or on the iRhom2/ADAM17 interaction (Figures 3B–3E and S3A). Combining all four mutations (EEHA) slightly increased ADAM17 shedding activity and reduced interaction with mature ADAM17 (Figures S4A–S4D). Similarly, mutations of L409 and S419 on interface 4 showed limited effect on ADAM17 activity or maturation (Figures 3B, 3C, and S3A) or on the iRhom2/ADAM17 complex (Figure 3F), even when combined (Figures S4E–S4H). In light of these minor effects, we did not further explore mutants at interfaces 2 and 4.

By contrast, mutations that disrupt interface 3 (D475R and D475A) (Figure 4A) caused a substantial increase in ADAM17 shedding activity in unstimulated cells (Figure 4B). This basal shedding activity of ADAM17 was seen not only with AREG but also with other substrates, including TNF and transforming growth factor alpha (TGFα) (Figures S3F and S3G). Importantly, these mutations had no effect on the activity of ADAM10 (Figure S3H). D475R also caused loss of mature ADAM17 protein (Figure 4C), which appears contradictory to the increased shedding activity seen with this iRhom2 mutant. We hypothesized that ADAM17 activity might trigger its degradation, leading to reduced levels of the mature enzyme, a recurring theme in many signaling systems. Indeed, treatment with proteasome inhibitors (bortezomib, BTZ) or lysosomal degradation inhibitor (bafilomycin A1, BafA1) each rescued the presence of mature ADAM17 in cells expressing iRhom2 D475R (Figures 4D and 4E), albeit BafA1 had a milder effect. These observations demonstrate that iRhom2 D475R does support the maturation of

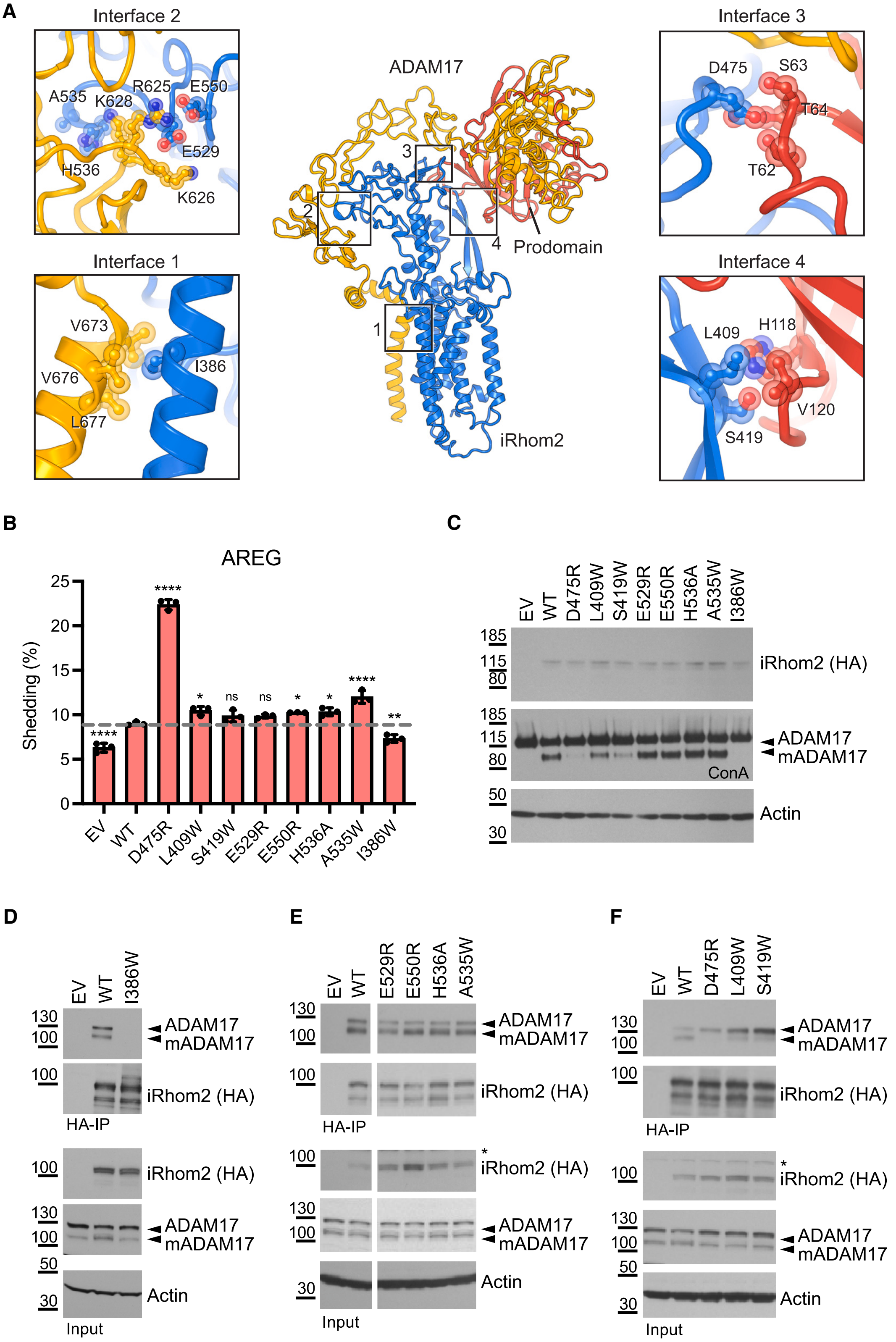

(legend on next page)

ADAM17 and that the absence of mature ADAM17 is a consequence of subsequent activity-dependent degradation. In further support of this conclusion, treatment with inhibitor GW280264X (which inhibits both ADAM17 and ADAM10) but not with the inhibitor GI254023X (which mostly inhibits ADAM10) fully rescued the presence of mature ADAM17 in iRhom2 D475R cells (Figures 4B and 4C).

In summary, functional analyses of the interfaces between iRhom2 and ADAM17 reveal two classes of major regulatory interactions. The first occurs between TMDs within the membrane bilayer, and this establishes the complex that is competent for trafficking and maturation. The second class of interaction takes place between the iRhom2 IRHD (particularly around residue D475) and the ADAM17 prodomain. This unexpected interaction inhibits the activity of ADAM17, suppressing shedding in unstimulated cells.

ADAM17 activation is regulated by iRhom2

We next studied the mechanism by which ADAM17 is activated. The prevailing model[15] posits that furin cleavage at the junction between the inhibitory prodomain and the protease domain of ADAM17 results in the detachment of the prodomain (Figure 2B), thus generating mature, active protease. iRhom2 had not previously been implicated in this process, but our structural and functional analyses encouraged us to reassess the relationship between furin cleavage and ADAM17 activation and the role of iRhom2. We coexpressed the ADAM17 prodomain (residues 1–214) in *trans* with mature ADAM17 (residues 215–824) and iRhom2 and determined the structure of this complex. We found that its conformation is almost identical to the inactive sheddase complex containing intact, full-length ADAM17 (Figure 5A). The non-covalently linked prodomain remains attached to inhibit ADAM17. This clearly indicates that interactions with iRhom2 and the rest of ADAM17 can hold the prodomain in place even after furin cleavage, restraining unstimulated activity of ADAM17.

To confirm that the prodomain is also retained *in vivo*, we expressed full-length ADAM17 and sought to detect the tripartite complex (ADAM17 prodomain/mature ADAM17/iRhom2) in cells. This proved to be challenging, presumably because the complex is transient and short lived and because, at steady state, most ADAM17 is in its immature form in the ER.[46] Significantly, when a cysteine bridge is introduced at the D475 interface between iRhom2 and ADAM17, or nearby (at S477), the furin-cleaved ADAM17 prodomain was retained in the complex (Figure 5B). A cysteine link introduced further from the D475 (at V416) was much less effective at prodomain retention (Figure 5B). To investigate further prodomain retention, we took advantage of the ADAM17 mutation R58A, which slows down prodomain release.[47] In cells expressing ADAM17 R58A, the level of the tripartite complex was increased to a level where it could be reliably detected. In that context, we found that disrupting interface 3 by mutating iRhom2 D475 substantially reduced the amount of prodomain in the complex (Figure 5C). These results provide experimental support for the interpretation that iRhom2 participates in retaining the furin-cleaved prodomain in cells.

Our structural and functional results highlight the importance of prodomain retention by iRhom2 and suggest that the interaction with IRHD prevents unregulated ADAM17 shedding activity in unstimulated cells. We thus proceeded to explore what occurs when cells are stimulated to initiate shedding. We treated cells with phorbol-12-myristate-13-acetate (PMA), a potent activator of ADAM17 sheddase activity.[48] Remarkably, within 15 min of PMA treatment, we observed a substantial loss of the prodomain from the tripartite complex (Figure 5D). This finding indicates that the levels of prodomain retention can be dynamically adjusted upon stimulation, thereby modulating ADAM17 activity.

To provide further structural insights into the activation of ADAM17, we sought to determine the structure of the mature, active sheddase complex. We coexpressed mature ADAM17 (residues 215–824), iRhom2, and FRMD8. This purified complex was active and capable of cleaving a model substrate (Figure S5A). Cryo-EM imaging and reference-free two-dimensional (2D) classification of the mature complex revealed a diffuse density for ADAM17 (Figure 5E), indicating a high degree of flexibility that precluded high-resolution structural analysis. This observation is consistent with the importance of prodomain retention in regulating the complex beyond its established role of inhibiting the catalytic site. Upon release of the prodomain, the sheddase complex becomes more flexible, and this structural transition is correlated with ADAM17 activation.

To stabilize the active complex, we generated mature ADAM17-iRhom2 fusion constructs in which the C terminus of iRhom2 was fused to the N terminus of mature ADAM17 (Figure S5B). Similar to the WT mature complex, these fusion

Figure 3. Dissecting the interfaces between iRhom2 and ADAM17

(A) Four major interfaces between iRhom2 and ADAM17 are highlighted. Selected residues comprising the interfaces (within 5 Å distance) are shown in ball-and-stick representation. The ADAM17 prodomain is colored red, whereas the rest of ADAM17 is colored yellow. For highlighted side chains, we colored their oxygen atoms in red, nitrogen atoms in purple blue, and carbon atoms the same color as the main chain.

(B) iRhom1/2 DKO HEK cells were transfected with empty vector (EV) or different iRhom2 single point mutants together with the ADAM17 substrate alkaline phosphatase (AP)-tagged amphiregulin (AREG). The growth medium was collected overnight and used for the AP-shedding assay. Substrate shedding (%), which represents ADAM17 shedding activity, was calculated by dividing the level of released alkaline phosphatase in the medium by the total alkaline phosphatase level. Error bars represent standard deviations ($n = 3$, three transfectants). A Dunnett's test is performed by computing a Student's t statistic for each transfection condition, and the statistic compares the transfection control (EV) and all iRhom2 mutants (D475R, L409W, S419W, E529R, E550R, H536A, A535W, and I386W) to the WT iRhom2 condition. ****$p < 0.0001$, **$p < 0.01$, *$p < 0.05$; ns, not significant.

(C) Concanavalin A (ConA) enrichment was performed to the lysates from the AREG shedding assay to quantify levels in cells of the iRhom2 mutants as well as full-length, immature (ADAM17), and mature ADAM17 (mADAM17).

(D–F) Cell lysates and anti-hemagglutinin (HA) immunoprecipitates were blotted for endogenous ADAM17, HA (iRhom2), and actin. In (E), EV and WT conditions are on the same immunoblot as mutant conditions, with superfluous lanes removed to make comparison easier. *indicates non-specific signal. Data are representative of three independent experiments (B–F).

See also Figures S3 and S4 an Table S1.

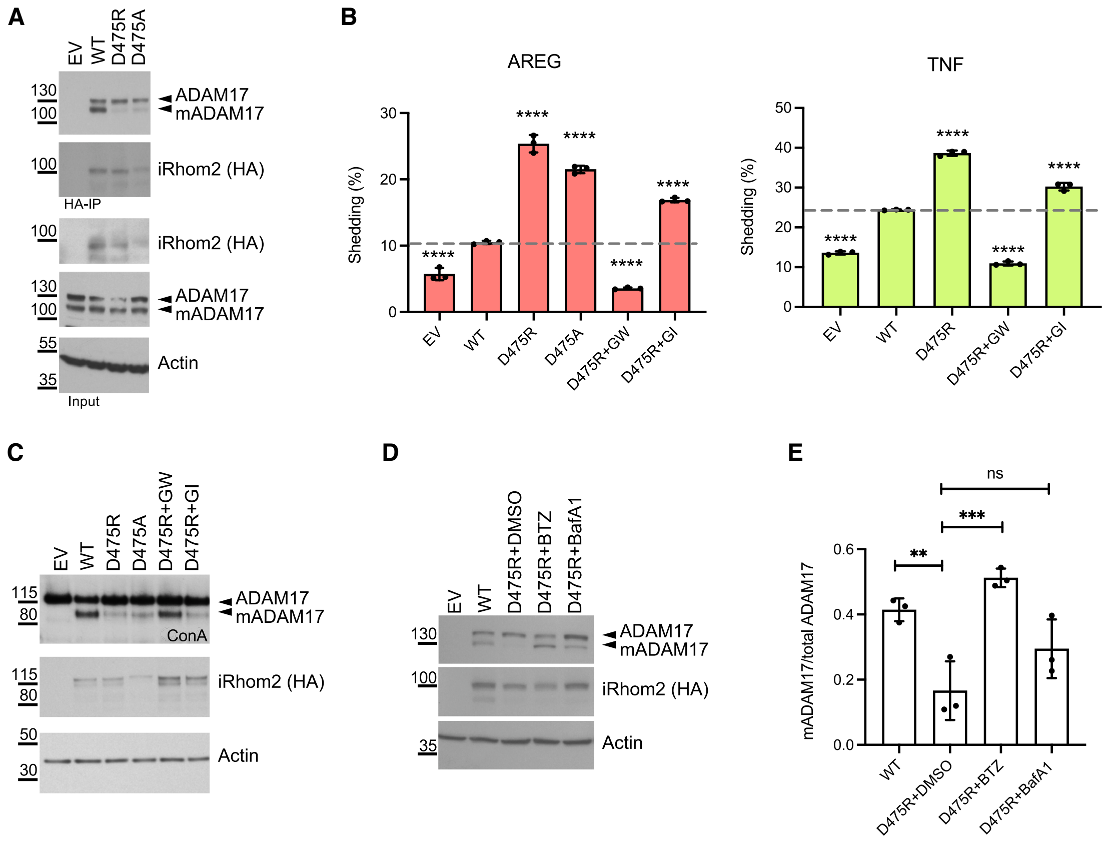

Figure 4. Mutations at D475 in iRhom2 drive unstimulated ADAM17 activation and degradation

(A) HA-based immunoprecipitates and lysates were blotted for ADAM17, HA (iRhom2), and actin.

(B) iRhom1/2 DKO HEK cells were transfected with different iRhom2 variants together with AP-tagged AREG or TNF as ADAM17 substrates. Medium was collected overnight and 2 μM GW280264X (GW) or GI254023X (GI) were used when indicated. Error bars represent standard deviations (n = 3, three transfectants). A Dunnett's test is performed by computing a Student's t statistic for each transfection condition compared with the WT iRhom2 condition. ****p < 0.0001.

(C) Concanavalin A (ConA) enrichment was performed to the lysates from the AREG shedding assay.

(D) Western blots of iRhom1/2 DKO HEK293 cells transfected with iRhom2 mutants together with wild-type (WT) mScarlet-tagged ADAM17. Cells were treated with bortezomib (BTZ, 1 μM), or bafilomycin A1 (BafA1, 1 μM) for 12 h before harvesting. DMSO was used as a solvent control.

(E) Quantifications of the western blots from three independent experiments of (D) using ImageJ. Error bars represent standard deviations (n = 3, three independent experiments). A Dunnett's test is performed by computing a Student's t statistic for each transfection condition compared with D475R+DMSO condition. ***p < 0.0001, **p < 0.01; ns, not significant. Data are representative of three independent experiments (A–D).

See also Figure S3.

constructs were proteolytically active and could be inhibited by an ADAM17 protease inhibitor (Figure S5B, upper panel); iRhom2-ADAM17 fusion protein also supported PMA-stimulated shedding of AREG in cells (Figure S5B, lower panel). We were able to determine structures of the fusion constructs to 2.3 Å resolution (Figures S5C–S5G). The protease domain of ADAM17 remains highly dynamic and only displays as a blob-like, low-resolution feature (Figure 5F). Nevertheless, it is clear that there are substantial conformational changes in the protease domain compared with the inactive complex, with a rotation of at least 30° upon activation. The rest of the extracellular domain of ADAM17 also undergoes noticeable displacements (Figure 5G). Thus, in the mature sheddase complex, it appears that the interactions between ADAM17 and iRhom2 extracellular regions become transient, allowing ADAM17 to adopt multiple states. The conformations that we observed likely represent some of these transient states. We propose that the large conformational space sampled by the protease domain enables it to approach its diverse substrates, which have a range of shapes and sizes. The dynamic nature of mature ADAM17 may be crucial for its biological function as a versatile protease capable of effectively cleaving a multitude of substrates.

DISCUSSION

ADAM17 is reported to shed more than eighty substrates from the cell surface, including growth factors and the cytokine TNF, the primary trigger of inflammation.[4] In addition to TNF, its most studied substrate, ADAM17 also cleaves macrophage colony-stimulating factor (M-CSF) and TNF receptor 1

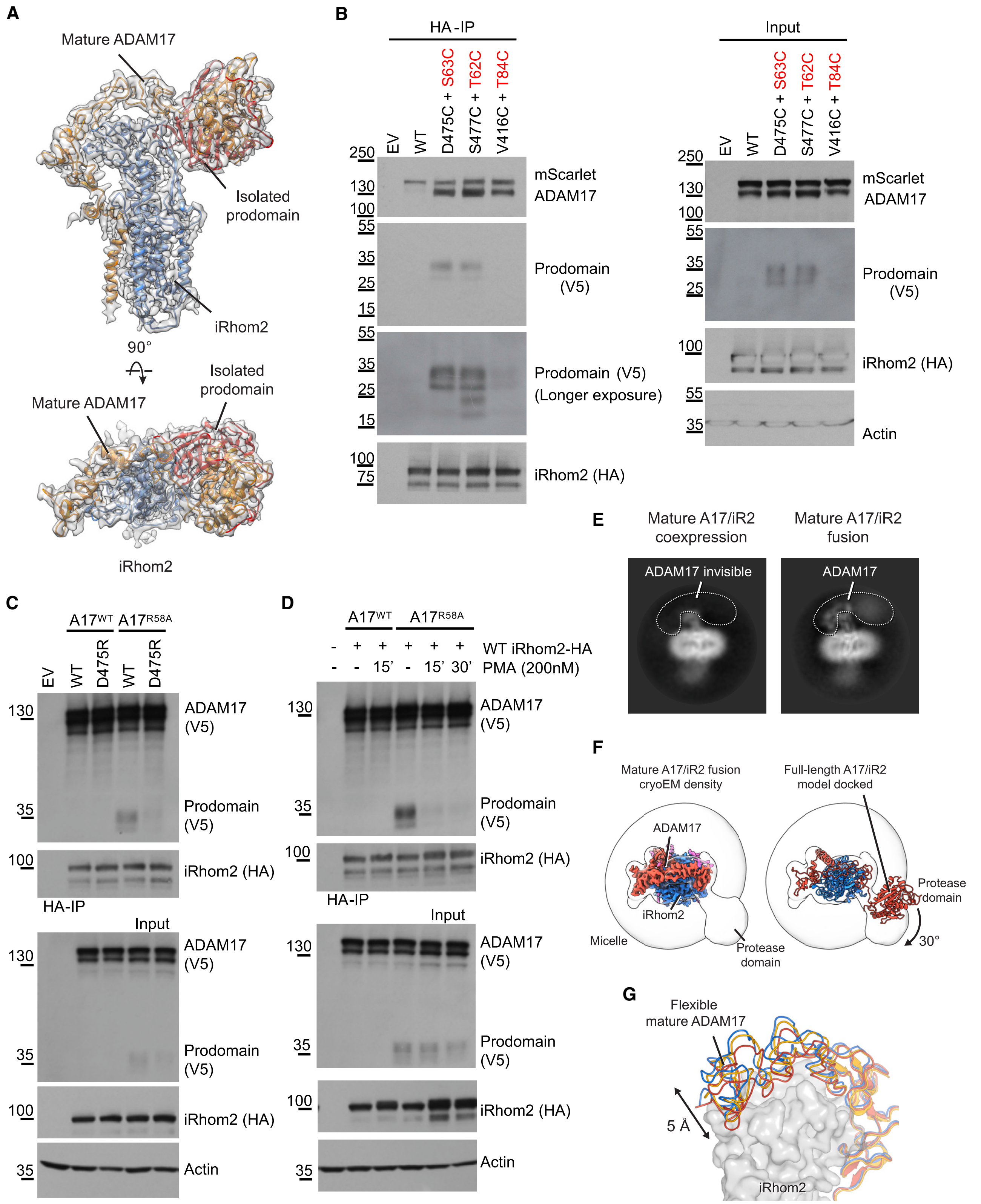

(legend on next page)

(TNFR1), thus playing essential immunomodulatory roles in establishing the balance between inflammation and host defense.[49,50] Recently, it has been reported that ADAM17-mediated shedding is also involved in the pathogenesis of COVID19.[51] Unregulated ADAM17 activity leads to unwarranted release of cytokines and other substrates central to the pathogenesis of multiple diseases, so it must be precisely controlled. Our structural and functional analyses of the ADAM17/iRhom2 sheddase complex reveal the mechanisms underlying its regulation and activity in molecular detail. Overall, we describe how iRhom2, a member of the rhomboid-like superfamily, has evolved to control multiple stages of the ADAM17 life cycle, including a previously unknown inhibitory role, to ensure precise regulation of inflammatory and growth factor signaling.

It was expected that iRhoms, which evolved from rhomboid proteases, would interact with ADAM17 in a manner similar to rhomboid proteases engaging their substrates.[24,25,37] However, our data show this expectation was unfounded. Instead of using the equivalent interface as the rhomboid protease substrate binding site (between TMD2 and TMD5), iRhom2 interacts with ADAM17 via an interface formed primarily by TMD1. This finding challenges the simplest evolutionary scenario, wherein the pseudoprotease/client interaction has evolved directly from the protease/substrate interaction. However, it is possible that iRhom2 can simultaneously bind to ADAM17 and other membrane clients such as ADAM17 substrates. In this scenario, the TMD2-TMD5 interface might remain functionally significant.

We have developed a mechanistic model that explains how iRhom2 regulates ADAM17. The interaction between the iRhom2 TMD1 and the ADAM17 TMD is essential for the earliest-known regulatory step—the trafficking of the complex from the ER to the Golgi apparatus. Loss of this interaction prevents ADAM17 trafficking and maturation, leading to a phenotype that resembles the complete loss of iRhom2 (Figures 3B–3D, also Siggs et al.[43]). This TMD-based interaction can be considered primary, reflecting the core function of rhomboid-like proteins, and we expect it to be retained throughout the existence of the ADAM17/iRhom2 complex.

After furin cleaves the peptide bond between the ADAM17 prodomain and the active enzyme, the prodomain remains associated with the ADAM17/iRhom2 complex via its unexpected interaction with the IRHD of iRhom2. This tripartite complex is transient and short lived, but critical for preventing premature and unregulated shedding activity, as evidenced by the elevated unstimulated shedding observed in the iRhom2 D475 mutants (Figures 3B and 4B).

Post maturation, ADAM17 activity can be further stimulated by phosphorylation of the iRhom2 cytoplasmic tail and 14-3-3 binding, which induces a conformational change in iRhom2, weakening its interaction with ADAM17 and leading to ADAM17 activation.[18,19] The molecular details of this intracellular regulatory step remain unknown, but our data show that PMA can reduce the level of the prodomain associated with the sheddase complex. We therefore speculate that cytoplasmic signals are propagated to the IRHD in a manner that promotes the dissociation of the tripartite complex.

Our structural data show that, in addition to competitively inhibiting the active site of ADAM17, the prodomain also constrains the flexibility of the mature protease. In the absence of the prodomain, there is strikingly increased mobility of the protease and other extracellular domains of the enzyme. We propose that the conformational freedom provided by loss of the prodomain contributes to the ability of ADAM17 to cleave its multiple substrates in their juxtamembrane domains. Recently, AlphaFold-predicted models of the ADAM17/iRhom2 complex have been generated[52]; while the overall architecture of the computational models was similar to our cryo-EM structures, there are noticeable distortions and domain rearrangements, with a root-mean-square deviation up to 2.7 Å. Moreover, in the predicted models, the protease domain of mature ADAM17 remains associated with iRhom2 IRHD, whereas our data indicate that these domains dissociate to allow the conformational flexibility of mature ADAM17.

Finally, we also detected a modest elevation of sheddase activity when the EEHA motif in iRhom2 at interface 2 was mutated (Figures S4A–S4D), indicating that this interface also contributes to restraining ADAM17 protease activity. Intriguingly, this interface overlaps with a previously identified phosphatidylserine binding site in ADAM17, which was proposed to mediate ADAM17 activation.[14,53]

Figure 5. Activation of ADAM17 and its regulation by iRhom2

(A) Cryo-EM density (gray) and the structure of the tripartite complex (isolated ADAM17 prodomain, mature ADAM17, and iRhom2).

(B) iRhom1/2 DKO HEK cells were transfected with either empty vector (EV), WT, or mutant HA-tagged iRhom2, and WT or mutant ADAM17 (indicated in red, with prodomain tagged with V5 and cytoplasmic domain tagged with mScarlet). Anti-HA immunoprecipitation (HA-IP) was performed to capture the ADAM17 prodomain in complex with iRhom2. Samples were blotted for mScarlet (ADAM17), prodomain (V5), and HA (iRhom2). A reducing sample buffer was used to separate the prodomain from the complex for analysis with western blots.

(C) iRhom1/2 DKO HEK cells were transfected with WT or D475R iRhom2 together with WT or R58A ADAM17, with a V5 tag inserted before the furin cleavage site and an mScarlet tag at the C terminus. HA-based immunoprecipitates and lysates were blotted with V5 (full-length ADAM17 and its prodomain), HA (iRhom2), and actin.

(D) iRhom1/2 DKO HEK cells were transfected with WT iRhom2 together with WT or R58A ADAM17 constructs. Cells were treated with 200 nM PMA for the indicated time (15 mins or 30 min) before harvesting. DMSO was used as solvent control.

(E) Two-dimensional class averages of the mature ADAM17 complex and mature ADAM17/iRhom2 fusion.

(F) Structural analyses of the mature ADAM17/iRhom2 fusion. To show the flexible protease domain, the density map is also lowpass filtered and contoured at a lower threshold (white). On the right, the structure of the full-length ADAM17/iRhom2 complex is docked into the density to demonstrate the movement of the protease domain. For clarity, the prodomain structure is not shown.

(G) Movements of the mature ADAM17 extracellular domain. Structures of the full-length ADAM17/iRhom2 and two mature ADAM17 fusions are superimposed.

Data are representative of three independent experiments (B–D).

See also Figure S5.

CellPress
OPEN ACCESS

Table 1. Cryo-EM data collection, processing, and refinement statistics

Structure	ADAM17/iRhom2	Prodomain/mature ADAM17/iRhom2	Mature ADAM17/iRhom2 (5 aa linker fusion)	Mature ADAM17/iRhom2 (3 aa linker fusion)
PDB	8SNL	8SNM	8SNN	8SNO
EMDB	EMD-40628	EMD-40629	EMD-40630	EMD-40631
Data collection/processing				
Magnification	105,000×	63,000×	105,000×	105,000×
Voltage (kV)	300	200	300	300
Pixel size (Å)	0.826	1.314	0.826	0.826
Defocus range (μm)	0.6–1.6	0.6–1.6	0.6–1.6	0.6–1.6
Electron exposure (e^{-}/Å2)	79.8	65.4	67.8	66.6
Symmetry imposed	C1	C1	C1	C1
Initial particles (no)	~5.6 millions	~1.7 millions	~7.3 millions	~5.2 millions
Final particles (no)	229,986	152,751	774,221	325,764
Map resolution (Å)	2.78	3.84	2.32	2.78
FSC threshold	0.143	0.143	0.143	0.143
Map resolution range (Å)	42–2.4	40–3.3	39–2.0	43–2.4
Refinement				
Model resolution (Å)	3.1	4.0	2.4	2.9
FSC threshold	0.5	0.5	0.5	0.5
Map sharpening B-factor (Å2)	−65.3	−151.7	−67.6	−63.7
Model composition				
Non-hydrogen atoms	9,098	8,986	5,685	5,685
Protein residues	1,147	1,134	718	718
Ligand	2	2	1	1
B-factors (Å2)				
Protein	75.22	73.14	44.61	52.97
Ligand	76.91	83.32	71.31	70.85
R.m.s. deviations				
Bond lengths (Å)	0.004	0.004	0.003	0.004
Bond angles (°)	0.738	0.752	0.649	0.712
Validation				
MolProbity score	1.93	1.95	1.87	1.87
Clashscore	10.55	11.26	8.75	9.47
Rotamers outliers (%)	0.00	0.00	0.00	0.00
Ramachandran plot (%)				
Favored	94.30	94.40	93.98	94.54
Allowed	5.70	5.60	6.02	5.46
Outliers	0.00	0.00	0.00	0.00

The ADAM17/iRhom2 complex is strikingly different from the recently reported structure of ADAM10 in complex with its regulator Tspan15.[39] In that case, the transmembrane regulator holds the enzyme active site in an open conformation and acts as a molecular ruler, ensuring that substrates are cleaved at the appropriate distance from the membrane surface. Notably, unlike ADAM17 and iRhom2, there appear to be no stable transmembrane interactions between ADAM10 and Tspan15, nor was there any reported evidence for Tspan15 participating in regulating prodomain association. It would therefore appear that within the broader theme of signal-regulating membrane proteins, multiple control mechanisms have evolved. This potentially opens the door to designing selective therapeutic strategies that specifically target ADAM17, a historical challenge in the development of drugs targeting ADAM family proteases.

Our study has established a framework for understanding how iRhom2 regulates ADAM17, but there are still important unanswered questions. For example, residue R58 of ADAM17 has been proposed as another cleavage site involved in ADAM17 maturation.[47] Although ADAM17 R58 does not interact directly with iRhom2 D475, these two residues are in close proximity, raising the possibility that iRhom2 D475 might allosterically

regulate cleavage at ADAM17 R58. Another open question is how iRhom2 affects ADAM17 substrate selectivity.[30] Obtaining structures of the sheddase complex complete with a substrate *in situ* would provide valuable insights into substrate recognition and processing. Finally, our structures do not resolve the intracellular domains of ADAM17 or iRhom2 and thus cannot inform on regulatory events within the cytoplasm. For example, it is not known how phosphorylation and 14-3-3 binding transmit conformational changes to the extracellular side[18,19] or how FRMD8 binding stabilizes iRhom2.[20,21] These knowledge gaps present exciting opportunities for further research. Nevertheless, the insights gained from our structures of the ADAM17/iRhom2 complex, including its regulatory mechanisms, provide a mechanistic basis for the development of future therapeutics to target ADAM17-dependent shedding in inflammatory diseases and cancer.

Limitations of the study

In addition to the binding of FRMD8, phosphorylation on the N terminus of iRhom2, followed by the recruitment of 14-3-3 proteins, is known to modulate ADAM17 activation.[18,19] However, due to the flexibility of the cytoplasmic domains of the iRhom2/ADAM17 complex, we were unable to resolve the structure of the N terminus of iRhom2 and its binding partner FRMD8. The mechanisms by which 14-3-3 and FRMD8 interactions regulate ADAM17 therefore remain to be investigated. Furthermore, due to the dynamic nature of the mature sheddase complex, we used fusion constructs to probe the structural changes upon activation. Orthogonal approaches would help to reveal the conformational landscape of the active complex under physiological conditions.

Another limitation, which needs to be addressed in the future, arises at the cell biological level. It is not yet clear where exactly in the secretory pathway the maturation events of prodomain cleavage and iRhom2-dependent retention occur. Understanding this will lead to a more detailed understanding of the regulation of cytokine and growth factor shedding. Additionally, we have not explored in detail the comparative structures, functions, and regulatory mechanisms of iRhom2 and iRhom1. Despite their similarities, the two iRhoms in humans are known to perform distinct functions, are expressed in different cell types, and have markedly different implications for diseases.[54,55]

STAR★METHODS

Detailed methods are provided in the online version of this paper and include the following:

- KEY RESOURCES TABLE
- RESOURCE AVAILABILITY
  - Lead contact
  - Materials availability
  - Data and code availability
- EXPERIMENTAL MODEL AND STUDY PARTICIPANT DETAILS
  - Bacterial strains
  - Cell lines
- METHOD DETAILS
  - Construct design
  - Protein expression and purification
  - Cryo-EM sample preparation and data acquisition
  - Cryo-EM image processing
  - Model building and refinement
  - DNA transfection
  - Co-immunoprecipitation
  - Concanavalin A enrichment
  - SDS-PAGE and western blotting
  - Alkaline phosphatase—Shedding assay
  - Peptide substrate cleavage assay
- QUANTIFICATION AND STATISTICAL ANALYSIS

SUPPLEMENTAL INFORMATION

Supplemental information can be found online at https://doi.org/10.1016/j.molcel.2024.04.025.

ACKNOWLEDGMENTS

We thank scientists in the Cryo-EM Center of St. Jude Children's Research Hospital for their support in data collection. We thank the Freeman lab members for contributions during the course of the project and Dr. Adam Grieve for feedback on the manuscript. We thank Dr. Ines Chen for her help with editing the manuscript. This work was supported by NIH R01 GM143282 to C.-H.L., ALSAC to C.-H.L., and Wellcome Trust Investigator awards to M.F. (101035/Z/13/Z and 220887/Z/20/Z).

AUTHOR CONTRIBUTIONS

F.L. designed, performed, and analyzed cellular functional experiments. H.Z. designed, performed, and analyzed biochemical and structural experiments. Y.D. performed structural experiments. Y.W. assisted in cell cultures. C.-H.L. and M.F. conceived the research and supervised the project. F.L., C.-H.L., and M.F. wrote the manuscript. All authors contributed to manuscript preparation.

DECLARATION OF INTERESTS

The authors declare no competing financial interests.

Received: August 29, 2023
Revised: February 9, 2024
Accepted: April 30, 2024
Published: May 22, 2024

REFERENCES

1. Clark, P. (2014). Protease-mediated ectodomain shedding. Thorax *69*, 682–684. https://doi.org/10.1136/thoraxjnl-2013-204403.
2. Lichtenthaler, S.F., Lemberg, M.K., and Fluhrer, R. (2018). Proteolytic ectodomain shedding of membrane proteins in mammals—hardware, concepts, and recent developments. EMBO J. *37*, e99456. https://doi.org/10.15252/embj.201899456.
3. Hayashida, K., Bartlett, A.H., Chen, Y., and Park, P.W. (2010). Molecular and Cellular Mechanisms of Ectodomain Shedding. Anat. Rec. (Hoboken) *293*, 925–937. https://doi.org/10.1002/ar.20757.
4. Zunke, F., and Rose-John, S. (2017). The shedding protease ADAM17: Physiology and pathophysiology. Biochim. Biophys. Acta Mol. Cell Res. *1864*, 2059–2070. https://doi.org/10.1016/j.bbamcr.2017.07.001.
5. Schumacher, N., and Rose-John, S. (2022). ADAM17 orchestrates Interleukin-6, TNFα and EGF-R signaling in inflammation and cancer. Biochim. Biophys. Acta Mol. Cell Res. *1869*, 119141. https://doi.org/10.1016/j.bbamcr.2021.119141.
6. Calligaris, M., Cuffaro, D., Bonelli, S., Spanò, D.P., Rossello, A., Nuti, E., and Scilabra, S.D. (2021). Strategies to Target ADAM17 in Disease: From Its Discovery to the iRhom Revolution. Molecules *26*, 944. https://doi.org/10.3390/MOLECULES26040944.

CellPress
OPEN ACCESS

7. Maskos, K., Fernandez-Catalan, C., Huber, R., Bourenkov, G.P., Bartunik, H., Ellestad, G.A., Reddy, P., Wolfson, M.F., Rauch, C.T., Castner, B.J., et al. (1998). Crystal structure of the catalytic domain of human tumor necrosis factor-α-converting enzyme. Proc. Natl. Acad. Sci. USA *95*, 3408–3412. https://doi.org/10.1073/pnas.95.7.3408.

8. Düsterhöft, S., Jung, S., Hung, C.-W., Tholey, A., Sönnichsen, F.D., Grötzinger, J., and Lorenzen, I. (2013). Membrane-Proximal Domain of a Disintegrin and Metalloprotease-17 Represents the Putative Molecular Switch of Its Shedding Activity Operated by Protein-disulfide Isomerase. J. Am. Chem. Soc. *135*, 5776–5781. https://doi.org/10.1021/ja400340u.

9. Moss, M.L., Jin, S.L., Milla, M.E., Bickett, D.M., Burkhart, W., Carter, H.L., Chen, W.J., Clay, W.C., Didsbury, J.R., Hassler, D., et al. (1997). Cloning of a disintegrin metalloproteinase that processes precursor tumour-necrosis factor-α. Nature *385*, 733–736. https://doi.org/10.1038/385733a0.

10. Black, R.A., Rauch, C.T., Kozlosky, C.J., Peschon, J.J., Slack, J.L., Wolfson, M.F., Castner, B.J., Stocking, K.L., Reddy, P., Srinivasan, S., et al. (1997). A metalloproteinase disintegrin that releases tumour-necrosis factor-α from cells. Nature *385*, 729–733. https://doi.org/10.1038/385729a0.

11. Seegar, T.C., and Blacklow, S.C. (2019). Domain integration of ADAM family proteins: Emerging themes from structural studies. Exp. Biol. Med. (Maywood) *244*, 1510–1519. https://doi.org/10.1177/1535370219865901.

12. Grötzinger, J., Lorenzen, I., and Düsterhöft, S. (2017). Molecular insights into the multilayered regulation of ADAM17: The role of the extracellular region. Biochim. Biophys. Acta Mol. Cell Res. *1864*, 2088–2095. https://doi.org/10.1016/j.bbamcr.2017.05.024.

13. Düsterhöft, S., Höbel, K., Oldefest, M., Lokau, J., Waetzig, G.H., Chalaris, A., Garbers, C., Scheller, J., Rose-John, S., Lorenzen, I., and Grötzinger, J. (2014). A Disintegrin and Metalloprotease 17 Dynamic Interaction Sequence, the Sweet Tooth for the Human Interleukin 6 Receptor *. J. Biol. Chem. *289*, 16336–16348. https://doi.org/10.1074/jbc.M114.557322.

14. Sommer, A., Kordowski, F., Büch, J., Maretzky, T., Evers, A., Andrä, J., Düsterhöft, S., Michalek, M., Lorenzen, I., Somasundaram, P., et al. (2016). Phosphatidylserine exposure is required for ADAM17 sheddase function. Nat. Commun. *7*, 11523. https://doi.org/10.1038/ncomms11523.

15. Schlöndorff, J., Becherer, J.D., and Blobel, C.P. (2000). Intracellular maturation and localization of the tumour necrosis factor alpha convertase (TACE). Biochem. J. *347*, 131–138.

16. Adrain, C., Zettl, M., Christova, Y., Taylor, N., and Freeman, M. (2012). Tumor necrosis factor signaling requires iRhom2 to promote trafficking and activation of TACE. Science *335*, 225–228. https://doi.org/10.1126/SCIENCE.1214400/SUPPL_FILE/1214400.ADRAIN.SOM.PDF.

17. McIlwain, D.R., Lang, P.A., Maretzky, T., Hamada, K., Ohishi, K., Maney, S.K., Berger, T., Murthy, A., Duncan, G., Xu, H.C., et al. (2012). iRhom2 regulation of TACE controls TNF-mediated protection against Listeria and responses to LPS. Science *335*, 229–232. https://doi.org/10.1126/science.1214448.

18. Grieve, A.G., Xu, H., Künzel, U., Bambrough, P., Sieber, B., and Freeman, M. (2017). Phosphorylation of iRhom2 at the plasma membrane controls mammalian TACE-dependent inflammatory and growth factor signalling. eLife *6*, 1–22. https://doi.org/10.7554/eLife.23968.

19. Cavadas, M., Oikonomidi, I., Gaspar, C.J., Burbridge, E., Badenes, M., Félix, I., Bolado, A., Hu, T., Bileck, A., Gerner, C., et al. (2017). Phosphorylation of iRhom2 Controls Stimulated Proteolytic Shedding by the Metalloprotease ADAM17/TACE. Cell Rep. *21*, 745–757. https://doi.org/10.1016/j.celrep.2017.09.074.

20. Künzel, U., Grieve, A.G., Meng, Y., Sieber, B., Cowley, S.A., and Freeman, M. (2018). FRMD8 promotes inflammatory and growth factor signalling by stabilising the iRhom/ADAM17 sheddase complex. eLife *7*, e35012. https://doi.org/10.7554/eLife.35012.

21. Oikonomidi, I., Burbridge, E., Cavadas, M., Sullivan, G., Collis, B., Naegele, H., Clancy, D., Brezinova, J., Hu, T., Bileck, A., et al. (2018). iTAP, a novel iRhom interactor, controls TNF secretion by policing the stability of iRhom/TACE. eLife *7*, e35032. https://doi.org/10.7554/eLife.35032.

22. Sieber, B., Lu, F., Stribbling, S.M., Grieve, A.G., Ryan, A.J., and Freeman, M. (2022). iRhom2 regulates ERBB signalling to promote KRAS-driven tumour growth of lung cancer cells. J. Cell Sci. *135*, jcs259949. https://doi.org/10.1242/JCS.259949.

23. Lemberg, M.K., and Freeman, M. (2007). Functional and evolutionary implications of enhanced genomic analysis of rhomboid intramembrane proteases. Genome Res. *17*, 1634–1646. https://doi.org/10.1101/gr.6425307.

24. Freeman, M. (2014). The rhomboid-like superfamily: molecular mechanisms and biological roles. Annu. Rev. Cell Dev. Biol. *30*, 235–254. https://doi.org/10.1146/ANNUREV-CELLBIO-100913-012944.

25. Wu, Z., Yan, N., Feng, L., Oberstein, A., Yan, H., Baker, R.P., Gu, L., Jeffrey, P.D., Urban, S., and Shi, Y. (2006). Structural analysis of a rhomboid family intramembrane protease reveals a gating mechanism for substrate entry. Nat. Struct. Mol. Biol. *13*, 1084–1091. https://doi.org/10.1038/nsmb1179.

26. Baker, R.P., and Urban, S. (2015). Cytosolic extensions directly regulate a rhomboid protease by modulating substrate gating. Nature *523*, 101–105. https://doi.org/10.1038/nature14357.

27. Ben-Shem, A., Fass, D., and Bibi, E. (2007). Structural basis for intramembrane proteolysis by rhomboid serine proteases. Proc. Natl. Acad. Sci. USA *104*, 462–466. https://doi.org/10.1073/PNAS.0609773104/ASSET/13D4990C-D052-4AD8-923D-1FBCE5071D73/ASSETS/GRAPHIC/ZPQ0520646110004.JPEG.

28. Moin, S.M., and Urban, S. (2012). Membrane immersion allows rhomboid proteases to achieve specificity by reading transmembrane segment dynamics. eLife *1*, e00173. https://doi.org/10.7554/ELIFE.00173.

29. Düsterhöft, S., Kahveci-Türköz, S., Wozniak, J., Seifert, A., Kasparek, P., Ohm, H., Liu, S., Kopkanova, J., Lokau, J., Garbers, C., et al. (2021). The iRhom homology domain is indispensable for ADAM17-mediated TNFα and EGF receptor ligand release. Cell. Mol. Life Sci. *78*, 5015–5040. https://doi.org/10.1007/s00018-021-03845-3.

30. Maretzky, T., McIlwain, D.R., Issuree, P.D.A., Li, X., Malapeira, J., Amin, S., Lang, P.A., Mak, T.W., and Blobel, C.P. (2013). iRhom2 controls the substrate selectivity of stimulated ADAM17-dependent ectodomain shedding. Proc. Natl. Acad. Sci. USA *110*, 11433–11438. https://doi.org/10.1073/PNAS.1302553110/SUPPL_FILE/PNAS.201302553SI.PDF.

31. Dulloo, I., Muliyil, S., and Freeman, M. (2019). The molecular, cellular and pathophysiological roles of iRhom pseudoproteases. Open Biol. *9*, 190003. https://doi.org/10.1098/rsob.190003.

32. Holm, L. (2022). Dali server: structural unification of protein families. Nucleic Acids Res. *50*, W210–W215. https://doi.org/10.1093/nar/gkac387.

33. Jumper, J., Evans, R., Pritzel, A., Green, T., Figurnov, M., Ronneberger, O., Tunyasuvunakool, K., Bates, R., Žídek, A., Potapenko, A., et al. (2021). Highly accurate protein structure prediction with AlphaFold. Nature *596*, 583–589. https://doi.org/10.1038/s41586-021-03819-2.

34. Wang, Y., Zhang, Y., and Ha, Y. (2006). Crystal structure of a rhomboid family intramembrane protease. Nature *444*, 179–180. https://doi.org/10.1038/nature05255.

35. Rao, B., Li, S., Yao, D., Wang, Q., Xia, Y., Jia, Y., Shen, Y., and Cao, Y. (2021). The cryo-EM structure of an ERAD protein channel formed by tetrameric human Derlin-1. Sci. Adv. *7*, eabe8591. https://doi.org/10.1126/sciadv.abe8591.

36. Wu, X., Siggel, M., Ovchinnikov, S., Mi, W., Svetlov, V., Nudler, E., Liao, M., Hummer, G., and Rapoport, T.A. (2020). Structural basis of ER-associated protein degradation mediated by the Hrd1 ubiquitin ligase complex. Science *368*, eaaz2449. https://doi.org/10.1126/science.aaz2449.

37. Baker, R.P., Young, K., Feng, L., Shi, Y., and Urban, S. (2007). Enzymatic analysis of a rhomboid intramembrane protease implicates

transmembrane helix 5 as the lateral substrate gate. Proc. Natl. Acad. Sci. USA *104*, 8257–8262. https://doi.org/10.1073/pnas.0700814104.

38. Seegar, T.C.M., Killingsworth, L.B., Saha, N., Meyer, P.A., Patra, D., Zimmerman, B., Janes, P.W., Rubinstein, E., Nikolov, D.B., Skiniotis, G., et al. (2017). Structural Basis for Regulated Proteolysis by the α-Secretase ADAM10. Cell *171*, 1638–1648.e7. https://doi.org/10.1016/j.cell.2017.11.014.

39. Lipper, C.H., Egan, E.D., Gabriel, K.-H., and Blacklow, S.C. (2023). Structural basis for membrane-proximal proteolysis of substrates by ADAM10. Cell *186*, 3632–3641.e10. https://doi.org/10.1016/j.cell.2023.06.026.

40. Van Wart, H.E., and Birkedal-Hansen, H. (1990). The cysteine switch: a principle of regulation of metalloproteinase activity with potential applicability to the entire matrix metalloproteinase gene family. Proc. Natl. Acad. Sci. USA *87*, 5578–5582. https://doi.org/10.1073/pnas.87.14.5578.

41. Gonzales, P.E., Solomon, A., Miller, A.B., Leesnitzer, M.A., Sagi, I., and Milla, M.E. (2004). Inhibition of the Tumor Necrosis Factor-α-converting Enzyme by Its Pro Domain*. J. Biol. Chem. *279*, 31638–31645. https://doi.org/10.1074/jbc.M401311200.

42. Wong, E., Cohen, T., Romi, E., Levin, M., Peleg, Y., Arad, U., Yaron, A., Milla, M.E., and Sagi, I. (2016). Harnessing the natural inhibitory domain to control TNFα Converting Enzyme (TACE) activity in vivo. Sci. Rep. *6*, 35598. https://doi.org/10.1038/srep35598.

43. Siggs, O.M., Xiao, N., Wang, Y., Shi, H., Tomisato, W., Li, X., Xia, Y., and Beutler, B. (2012). iRhom2 is required for the secretion of mouse TNFα. Blood *119*, 5769–5771. https://doi.org/10.1182/BLOOD-2012-03-417949.

44. Li, X., Maretzky, T., Perez-Aguilar, J.M., Monette, S., Weskamp, G., Le Gall, S., Beutler, B., Weinstein, H., and Blobel, C.P. (2017). Structural modeling defines transmembrane residues in ADAM17 that are crucial for Rhbdf2-ADAM17-dependent proteolysis. J. Cell Sci. *130*, 868–878. https://doi.org/10.1242/JCS.196436/265306/AM/STRUCTURAL-MODELING-DEFINES-TRANSMEMBRANE-RESIDUES.

45. Tang, B., Li, X., Maretzky, T., Perez-Aguilar, J.M., McIlwain, D., Xie, Y., Zheng, Y., Mak, T.W., Weinstein, H., and Blobel, C.P. (2020). Substrate-selective protein ectodomain shedding by ADAM17 and iRhom2 depends on their juxtamembrane and transmembrane domains. FASEB J. *34*, 4956–4969. https://doi.org/10.1096/fj.201902649R.

46. Lorenzen, I., Lokau, J., Korpys, Y., Oldefest, M., Flynn, C.M., Künzel, U., Garbers, C., Freeman, M., Grötzinger, J., and Düsterhöft, S. (2016). Control of ADAM17 activity by regulation of its cellular localisation. Sci. Rep. *6*, 35067. https://doi.org/10.1038/srep35067.

47. Wong, E., Maretzky, T., Peleg, Y., Blobel, C.P., and Sagi, I. (2015). The Functional Maturation of A Disintegrin and Metalloproteinase (ADAM) 9, 10, and 17 Requires Processing at a Newly Identified Proprotein Convertase (PC) Cleavage Site. J. Biol. Chem. *290*, 12135–12146. https://doi.org/10.1074/jbc.M114.624072.

48. Horiuchi, K., Le Gall, S., Schulte, M., Yamaguchi, T., Reiss, K., Murphy, G., Toyama, Y., Hartmann, D., Saftig, P., and Blobel, C.P. (2007). Substrate Selectivity of Epidermal Growth Factor-Receptor Ligand Sheddases and their Regulation by Phorbol Esters and Calcium Influx. Mol. Biol. Cell *18*, 176–188. https://doi.org/10.1091/mbc.e06-01-0014.

49. Bolik, J., Krause, F., Stevanovic, M., Gandraß, M., Thomsen, I., Schacht, S.-S., Rieser, E., Müller, M., Schumacher, N., Fritsch, J., et al. (2022). Inhibition of ADAM17 impairs endothelial cell necroptosis and blocks metastasis. J. Exp. Med. *219*, e20201039. https://doi.org/10.1084/jem.20201039.

50. Tang, J., Frey, J.M., Wilson, C.L., Moncada-Pazos, A., Levet, C., Freeman, M., Rosenfeld, M.E., Stanley, E.R., Raines, E.W., and Bornfeldt, K.E. (2018). Neutrophil and Macrophage Cell Surface Colony-Stimulating Factor 1 Shed by ADAM17 Drives Mouse Macrophage Proliferation in Acute and Chronic Inflammation. Mol. Cell. Biol. *38*, e00103-18. https://doi.org/10.1128/MCB.00103-18.

51. Schreiber, B., Patel, A., and Verma, A. (2020). Shedding Light on COVID-19: ADAM17 the Missing Link? Am. J. Ther. *28*, e358–e360.

52. Kahveci-Türköz, S., Bläsius, K., Wozniak, J., Rinkens, C., Seifert, A., Kasparek, P., Ohm, H., Oltzen, S., Nieszporek, M., Schwarz, N., et al. (2023). A structural model of the iRhom–ADAM17 sheddase complex reveals functional insights into its trafficking and activity. Cell. Mol. Life Sci. *80*, 135. https://doi.org/10.1007/s00018-023-04783-y.

53. Veit, M., Ahrens, B., Seidel, J., Sommer, A., Bhakdi, S., and Reiss, K. (2019). Mutagenesis of the ADAM17-phosphatidylserine–binding motif leads to embryonic lethality in mice. Life Sci. Alliance *2*, e201900430. https://doi.org/10.26508/lsa.201900430.

54. Babendreyer, A., Rojas-González, D.M., Giese, A.A., Fellendorf, S., Düsterhöft, S., Mela, P., and Ludwig, A. (2020). Differential Induction of the ADAM17 Regulators iRhom1 and 2 in Endothelial Cells. Front. Cardiovasc. Med. *7*, 610344. https://doi.org/10.3389/FCVM.2020.610344/BIBTEX.

55. Christova, Y., Adrain, C., Bambrough, P., Ibrahim, A., and Freeman, M. (2013). Mammalian iRhoms have distinct physiological functions including an essential role in TACE regulation. EMBO Rep. *14*, 884–890. https://doi.org/10.1038/EMBOR.2013.128.

56. Schindelin, J., Arganda-Carreras, I., Frise, E., Kaynig, V., Longair, M., Pietzsch, T., Preibisch, S., Rueden, C., Saalfeld, S., Schmid, B., et al. (2012). Fiji: an open-source platform for biological-image analysis. Nat. Methods *9*, 676–682. https://doi.org/10.1038/nmeth.2019.

57. Zheng, S.Q., Palovcak, E., Armache, J.-P., Verba, K.A., Cheng, Y., and Agard, D.A. (2017). MotionCor2 - anisotropic correction of beam-induced motion for improved cryo-electron microscopy. Nat. Methods *14*, 331–332. https://doi.org/10.1038/nmeth.4193.

58. Punjani, A., Rubinstein, J.L., Fleet, D.J., and Brubaker, M.A. (2017). cryoSPARC: algorithms for rapid unsupervised cryo-EM structure determination. Nat. Methods *14*, 290–296. https://doi.org/10.1038/nmeth.4169.

59. Zivanov, J., Nakane, T., Forsberg, B.O., Kimanius, D., Hagen, W.J., Lindahl, E., and Scheres, S.H. (2018). New tools for automated high-resolution cryo-EM structure determination in RELION-3. eLife *7*, e42166. https://doi.org/10.7554/eLife.42166.

60. Emsley, P., Lohkamp, B., Scott, W.G., and Cowtan, K. (2010). Features and development of Coot. Acta Crystallogr. D Biol. Crystallogr. *66*, 486–501. https://doi.org/10.1107/S0907444910007493.

61. Afonine, P.V., Poon, B.K., Read, R.J., Sobolev, O.V., Terwilliger, T.C., Urzhumtsev, A., and Adams, P.D. (2018). Real-space refinement in PHENIX for cryo-EM and crystallography. Acta Crystallogr. D Struct. Biol. *74*, 531–544. https://doi.org/10.1107/S2059798318006551.

62. Chen, V.B., Arendall, W.B., Headd, J.J., Keedy, D.A., Immormino, R.M., Kapral, G.J., Murray, L.W., Richardson, J.S., and Richardson, D.C. (2010). MolProbity: all-atom structure validation for macromolecular crystallography. Acta Crystallogr. D Biol. Crystallogr. *66*, 12–21. https://doi.org/10.1107/S0907444909042073.

63. Pettersen, E.F., Goddard, T.D., Huang, C.C., Couch, G.S., Greenblatt, D.M., Meng, E.C., and Ferrin, T.E. (2004). UCSF Chimera–a visualization system for exploratory research and analysis. J. Comput. Chem. *25*, 1605–1612. https://doi.org/10.1002/jcc.20084.

64. Goddard, T.D., Huang, C.C., Meng, E.C., Pettersen, E.F., Couch, G.S., Morris, J.H., and Ferrin, T.E. (2018). UCSF ChimeraX: Meeting modern challenges in visualization and analysis. Protein Sci. *27*, 14–25. https://doi.org/10.1002/pro.3235.

65. Goehring, A., Lee, C.-H., Wang, K.H., Michel, J.C., Claxton, D.P., Baconguis, I., Althoff, T., Fischer, S., Garcia, K.C., and Gouaux, E. (2014). Screening and large-scale expression of membrane proteins in mammalian cells for structural studies. Nat. Protoc. *9*, 2574–2585. https://doi.org/10.1038/nprot.2014.173.

66. Rana, M.S., Wang, X., and Banerjee, A. (2018). An Improved Strategy for Fluorescent Tagging of Membrane Proteins for Overexpression and

CellPress
OPEN ACCESS

Purification in Mammalian Cells. Biochemistry *57*, 6741–6751. https://doi.org/10.1021/acs.biochem.8b01070.

67. Weissmann, F., Petzold, G., VanderLinden, R., Huis in ’t Veld, P.J., Brown, N.G., Lampert, F., Westermann, S., Stark, H., Schulman, B.A., and Peters, J.-M. (2016). biGBac enables rapid gene assembly for the expression of large multisubunit protein complexes. Proc. Natl. Acad. Sci. USA *113*, E2564–E2569. https://doi.org/10.1073/pnas.1604935113.

68. Punjani, A., Zhang, H., and Fleet, D.J. (2020). Non-uniform refinement: adaptive regularization improves single-particle cryo-EM reconstruction. Nat. Methods *17*, 1214–1221. https://doi.org/10.1038/s41592-020-00990-8.

STAR★METHODS

KEY RESOURCES TABLE

REAGENT or RESOURCE	SOURCE	IDENTIFIER
**Antibodies**		
Rabbit polyclonal anti-ADAM17	Abcam	Cat# ab39162; RRID:AB_722565
Mouse monoclonal anti-RFP	ChromoTek	Cat# 6g6-100; RRID:AB_2631395
Mouse monoclonal anti- β-actin	Santa Cruz Biotechnology	Cat# sc-47778; RRID:AB_626632
Rabbit monoclonal anti-V5-Tag (D3H8Q)	Cell Signaling Technology	Cat# 13202; RRID:AB_2687461
Rat monoclonal anti-HA-HRP, (clone 3F10)	Roche	Cat# 12013819001; RRID:AB_390917
Goat polyclonal anti-rabbit-HRP	Bio-Rad	Cat# 170-6515; RRID:AB_11125142
Horse polyclonal anti-mouse-HRP	Cell Signaling Technology	Cat# 7076; RRID:AB_330924
Mouse monoclonal anti-beta-actin-HRP	Sigma-Aldrich	Cat# A3854; RRID:AB_262011
**Bacterial and virus strains**		
Stellar™ Competent Cells (an *E. coli* HST08 strain)	Takara Bio	Cat# 636763
E. coli DH5α Competent Cells	GoldBio	Cat# CC-101-TR
E. coli DH10Bac Competent Cells	Thermo Fisher	Cat# 10361012
**Chemicals, peptides, and recombinant proteins**		
1,10-Phenanthroline	Sigma-Aldrich	Cat# 131377-25G
cOmplete™, EDTA-free proteasecocktail	Roche	Cat# 11873580001
GW280264X (GW)	Generon	Cat# AOB3632-5
GI254023X (GI)	Sigma-Aldrich	Cat# SML0789-5MG
DMEM high glucose medium	Sigma-Aldrich	Cat# D6429-500ML
L-glutamine 200 mM (100x)	Gibco	Cat# 25030-024
Penicillin-Streptomycin(P/S 100x)	Gibco	Cat# 15140–122
Fetal Bovine Serum (FBS)	Sigma-Aldrich	Cat# F9665-500ML
FuGene HD Transfection Reagent	Promega	Cat# E2312
PNPP tablet	Thermo Fisher Scientific	Cat# 34047
Diethanolamine Substrate Buffer 5X concentrate	Thermo Fisher Scientific	Cat# 34064
Bortezomib (BTZ)	Selleck Chemical	Cat# S1013
Bafilomycin A1	Santa Cruz Biotechnology	Cat# sc-201550A
Sf-900 III SFM medium	Gibco	Cat# 12658027
TransIT-Insect transfection reagent	Mirus	Cat# MIR 6100
Freestyle 293 expression medium	Gibco	Cat# 12338018
Sodium butyrate	Millipore-Sigma	Cat# 303410
Protease Inhibitor Cocktail	Medchemexpress	Cat# HY-K0010
Benzonase nuclease	Millipore-Sigma	Cat# E1014-25KU
n-Dodecyl-β-D-Maltopyranoside (DDM)	Anatrace	Cat# D310
Cholesteryl Hemisuccinate Tris Salt (CHS)	Anatrace	Cat# CH210
Digitonin	Thermo Scientific	Cat# 407560050
TCEP solution	Thermo Scientific	Cat# 77720
**Deposited data**		
Cryo-EM structure of ADAM17/iRhom2	This paper	PDB: 8SNL; EMDB: EMD-40628
Cryo-EM structure of prodomain/mature ADAM17/iRhom2	This paper	PDB: 8SNM; EMDB: EMD-40629
Cryo-EM structure of mature ADAM17/iRhom2 (5 aa linker fusion)	This paper	PDB: 8SNN; EMDB: EMD-40630
Cryo-EM structure of mature ADAM17/iRhom2 (3 aa linker fusion)	This paper	PDB: 8SNO; EMDB: EMD-40631

(*Continued on next page*)

Continued

REAGENT or RESOURCE	SOURCE	IDENTIFIER
**Experimental models: Cell lines**		
HEK293T cells	Laboratory of Matthew Freeman	RRID:CVCL_0063
HEK293T iRhom1/iRhom2double-knockout (DKO)	Künzel et al.[20]	N/A
Sf9 insect cell	ATCC	Cat# CRL-1711; RRID:CVCL_0549
HEK-293S GnTI⁻	ATCC	Cat# CRL-3022; RRID:CVCL_A785
**Recombinant DNA**		
pcDNA3.1	Thermo Fisher Scientific	Cat# V790-20
pcDNA3.1_hiR2_iso2_WT	This paper	N/A
pcDNA3.1_hiR2_iso2_L409W	This paper	N/A
pcDNA3.1_hiR2_iso2_S419W	This paper	N/A
pcDNA3.1_hiR2_iso2_L409W/S419W	This paper	N/A
pcDNA3.1_hiR2_iso2_D475R	This paper	N/A
pcDNA3.1_hiR2_iso2_D475A	This paper	N/A
pcDNA3.1_hiR2_iso2_E529R	This paper	N/A
pcDNA3.1_hiR2_iso2_E550R	This paper	N/A
pcDNA3.1_hiR2_iso2_H536A	This paper	N/A
pcDNA3.1_hiR2_iso2_A535W	This paper	N/A
pcDNA3.1_hiR2_iso2_ E529R/E550R	This paper	N/A
pcDNA3.1_hiR2_iso2_ H536A/A535W	This paper	N/A
pcDNA3.1_hiR2_iso2_ E529R/E550R/ H536A/A535W	This paper	N/A
pcDNA3.1_hiR2_iso2_I386W	This paper	N/A
pCHL_ADAM17opt_mScarletI ALFA tag	This paper	N/A
pCHL_NFLAG_proV5_ADAM17optFL_ mScarletI ALFA tag_WT	This paper	N/A
pCHL_NFLAG_proV5_ADAM17optFL_ mScarletI ALFA tag_R58A	This paper	N/A
pCHL_iRhom2_full length ADAM17	This paper	N/A
pCHL_iRhom2_mADAM17	This paper	N/A
pCHL_iRhom2_GGS_mADAM17	This paper	N/A
pCHL_iRhom2_GGGGS_mADAM17	This paper	N/A
pCHL_iRhom2_mADAM17 fusion	This paper	N/A
pcDNA3.1_hiR2_iso2_D475C	This paper	N/A
pcDNA3.1_hiR2_iso2_S477C	This paper	N/A
pcDNA3.1_hiR2_iso2_V416C	This paper	N/A
pCHL_NFLAG_proV5_ADAM17optFL_ mScarletI ALFA tag_S63C	This paper	N/A
pCHL_NFLAG_proV5_ADAM17optFL_ mScarletI ALFA tag_T62C	This paper	N/A
pCHL_NFLAG_proV5_ADAM17optFL_ mScarletI ALFA tag_T84C	This paper	N/A
**Software and algorithms**		
Prism 10	GraphPad	https://www.graphpad.com/features
Fiji	Schindelin et al.[56]	https://fiji.sc/
MotionCor2	Zheng et al.[57]	https://emcore.ucsf.edu/ucsf-motioncor2
cryoSPARC v3.3.1	Punjani et al.[58]	https://cryosparc.com/
RELION 3.1	Zivanov et al.[59]	https://www3.mrc-lmb.cam.ac.uk/relion/
Coot 0.9.6	Emsley et al.[60]	https://www2.mrc-lmb.cam.ac.uk/personal/pemsley/coot/

(*Continued on next page*)

Continued		
REAGENT or RESOURCE	SOURCE	IDENTIFIER
Phenix 1.16	Adams et al.[61]	http://www.phenix-online.org/
PyMOL 2.3.4	Schrödinger	https://pymol.org/2/
MolProbity	Williams et al.[62]	http://molprobity.biochem.duke.edu
UCSF Chimera 1.13	Pettersen et al.[63]	http://www.cgl.ucsf.edu/chimera
ChimeraX	Goddard et al.[64]	https://www.cgl.ucsf.edu/chimerax/
Other		
Pierce™ anti-HA magnetic beads	Thermo Fisher Scientific	Cat# 88837
Concanavalin A sepharose	Sigma-Aldrich	Cat# C9017-25ML

RESOURCE AVAILABILITY

Lead contact

Further information and requests for resources and reagents should be directed to and will be fulfilled by the lead contact, M.F. (matthew.freeman@path.ox.ac.uk).

Materials availability

Plasmid generated in this study will be distributed upon request.

Data and code availability

- The cryo-EM density maps have been deposited in the Electron Microscopy Data Bank under the accession numbers EMD-40628, EMD-40629, EMD-40630, and EMD-40631. Atomic coordinates for the atomic model have been deposited in the Protein Data Bank under the accession numbers 8SNL, 8SNM, 8SNN, 8SNO. All deposited datasets will be publicly available upon publication.
- This paper does not report original code.
- Any additional information required to reanalyze the data reported in this paper is available from the lead contact upon request.

EXPERIMENTAL MODEL AND STUDY PARTICIPANT DETAILS

Bacterial strains

Stellar competent cells (an *E. coli* HST08 strain), DH5α competent cells, or DH10Bac competent cells were used for molecular cloning and amplification of the recombinant plasmids. Bacteria were grown on LB broth with appropriate antibiotics.

Cell lines

Human embryonic kidney (HEK) 293T cells and iRhom1/iRhom2 double knockout (DKO) HEK 293T cells[20] were cultured in DMEM (Sigma-Aldrich) supplemented with 10% fetal bovine serum (FBS, Sigma-Aldrich), 2 mM L-Glutamine,100 U/ml penicillin and 100 μg/ml streptomycin (all Gibco). Cells were cultured at 37°C with 5% CO_2 in a humidified cell culture incubator and were split twice a week using TrypLE Express (Gibco). Sf9 cells were cultured in Sf-900 III SFM medium at 27°C. HEK293S GnTI$^-$ cells were cultured in Freestyle 293 expression medium at 37°C.

METHOD DETAILS

Construct design

For biochemical and structural studies, the complementary DNA encoding human ADAM17 (UniprotKB: P78536-1), iRhom2 (isoform 2, UniprotKB: Q6PJF5-2) or FRMD8 (UniprotKB: Q9BZ67-1) was individually cloned into the pEG BacMam vector.[65] For full-length ADAM17, the coding sequence is followed by a TEV protease cleavage site and a C-terminal mScarlet tag. For the ADAM17 prodomain, the coding sequence includes amino acid residues 1-214. For mature ADAM17, the coding sequence includes amino acid residues 215-824, followed by a TEV protease cleavage site and a C-terminal mScarlet tag. For iRhom2, a N-terminal mVenus tag and a 3C protease cleavage site are inserted before the coding sequence.[66] For ADAM17 and iRhom2 fusion constructs, the C terminus of iRhom2 is connected to the N terminus of mature ADAM17 by a GlySer linker (GGGGS or GGS). The coding sequence is followed by a 3C protease cleavage site and a C-terminal mVenus tag. The expression cassettes containing individual genes were amplified and assembled into the pBIG1a vector using biGBac method.[67] The multigene expression constructs containing full-length ADAM17/iRhom2/FRMD8, prodomain/mature ADAM17/iRhom2/FRMD8, mature ADAM17/iRhom2/FRMD8, iRhom2-GGGGS-mature ADAM17/FRMD8, or iRhom2-GGS-mature ADAM17/FRMD8 were used for large-scale protein expression.

For functional studies, iRhom2 isoform 2 (aa 51–79 missing) was constructed based on the human iRhom2 isoform 1 cDNA (NM_024599.2; Origene, SC122961)[20] and was cloned into pcDNA3.1(+) using In-Fusion HD Cloning Kit (Takara Bio, 639649) with 3x HA tag at the C terminus. Site-directed mutagenesis of the gene of interest was performed using the Cloned Pfu DNA polymerase AD (Agilent) according to the manufacturer's instructions. Single colonies were picked and extracted DNA was verified by Sanger sequencing (Source BioScience, UK).

Protein expression and purification

The complexes were expressed in HEK293S GnTI$^-$ cells. Baculoviruses were produced by transfecting Sf9 cells with the bacmids using TransIT (Mirus). After one or two rounds of amplification, viruses were used for cell transduction. When HEK293S GnTI$^-$ suspension cultures grown at 37 °C reached a density of ~3.5 ×10^6 cells/ml, baculoviruses (10 % v/v) were added to initiate transduction. After 10–12 hrs, 10 mM sodium butyrate was supplemented to the cultures and the culture temperature was shifted to 25 °C. Cells were collected at 60 hr post-transduction.

The cell pellet was resuspended using hypotonic buffer (10 mM NaCl, 1 mM $MgCl_2$, 20 mM Tris pH 8, 2 mg/ml iodoacetamide, 0.1 mM TCEP, benzonase, and protease inhibitors) for 20 min. The cell lysate was then spun at 39,800g for 30 mins to sediment crude membranes. The membrane pellet was mechanically homogenized and solubilized in extraction buffer (20 mM DDM, 4 mM CHS, 150 mM NaCl, 20 mM Tris pH 8, 2 mg/ml iodoacetamide, 0.1 mM TCEP, benzonase, and protease inhibitors) for 1.5 h. Solubilized membranes were clarified by centrifugation at 39,800g for 45 mins. The supernatant was applied to the GFP nanobody-coupled glyoxal agarose resin (this nanobody also binds to mVenus), which was subsequently washed with 10 column volumes of wash buffer A (0.05 % digitonin, 150 mM NaCl, 0.1 mM TCEP, 4 mM NaATP, 4 mM $MgCl_2$ and 20 mM Tris pH 8), followed by 7 column volumes of wash buffer B (0.05 % digitonin, 150 mM NaCl, 0.1 mM TCEP and 20 mM Tris pH 8). The washed resin was incubated with 3C protease overnight at a target protein to protease ratio of 40:1 (w/w) to cleave off mVenus and release the protein from the resin. The protein was eluted with wash buffer B, concentrated, and further purified by gel-filtration chromatography using a Superose 6 increase column equilibrated with SEC buffer (0.05 % digitonin, 150 mM NaCl, 0.1 mM TCEP, and 20 mM Tris pH 8). Peak fractions were pooled and concentrated for cryo-EM experiments or peptide cleavage assay.

Cryo-EM sample preparation and data acquisition

Protein samples were concentrated to ~7 mg/ml. Aliquots of 3.5 μl protein samples were applied to plasma-cleaned Quantifoil UltrAuFoil R1.2/1.3 300 mesh grids. After 25 s, the grids were blotted for 3 s and plunged into liquid ethane using a Vitrobot Mark IV (FEI) operated at 10 °C and 100% humidity. The grids were loaded onto a 300 kV Titan Krios or 200kV Talos Arctica transmission electron microscope for data collection. Raw movie stacks were recorded with a K3 camera at a physical pixel size of 0.826 Å on Krios and of 1.314 Å on Arctica. The nominal defocus range was 0.6–1.6 μm. The exposure time for each micrograph was 2-3 s on Krios and 5 s on Arctica, dose-fractionated into 60-70 frames with approximately 1 $e^-/Å^2$ per frame. Image acquisition parameters are summarized in Table 1.

Cryo-EM image processing

The image stacks were firstly gain-normalized and corrected for beam-induced motion using MotionCor2.[57] Defocus parameters were estimated from motion-corrected images using cryoSPARC3.[58] Micrographs not suitable for further analysis were removed by manual inspection. Particle picking (blob picker and template picker) and 2D classifications were done in cryoSPARC3 (Figures 5C andS1A). After 2-3 rounds of 2D classifications, selected particles were used for iterative 3D classifications including ab initio reconstructions and heterogeneous refinements to remove suboptimal particles. The best classes were then subjected to non-uniform refinements for 3D reconstructions.[68] The refined particles were subjected to Bayesian polishing in RELION 3.1.[59] The polished particles were imported into cryoSPARC3 where additional non-uniform refinements were performed (Figures 5C and S1A). The mask-corrected FSC curves were calculated in cryoSPARC3, and reported resolutions are based on the 0.143 criterion (Figures 5D and S1B). Local resolution estimations were performed in cryoSPARC3 (Figures 5F and S1D).

Model building and refinement

For ADAM17, structures of the protease domain (PDB: 1BKC), MPD (PDB: 2M2F), and other domains (predicted by AlphaFold[33]) were docked into cryo-EM density maps using Chimera.[63] For iRhom2, a predicted model was generated by AlphaFold and docked into density maps. The resulting model of the complex was then iteratively refined in Coot[60] and Phenix.[61] The structural model of full-length ADAM17 includes residues 28–203, and 219–698. The structural model of mature ADAM17 includes residues 477–703. The structural model of iRhom2 includes residues 337–827. Model validation was performed using Phenix and MolProbity.[62] Figures were prepared using PyMOL, Chimera, and ChimeraX.[64]

DNA transfection

FuGENE® HD (Promega) was used for transient DNA transfection, with a 4:1 ratio of transfection reagent (μl): DNA (μg), both of which were diluted in OptiMEM (Gibco).

Co-immunoprecipitation

Cells were washed with ice-cold PBS before lysis in Triton X-100 lysis buffer (1% Triton X-100, 150 mM NaCl, 50 mM Tris-HCl, pH 7.5) supplemented with EDTA-free complete protease inhibitor mix (Roche, 11873580001), and 10 mM 1,10-phenanthroline (Sigma-Aldrich, 131377–5G). Cell lysates were cleared by centrifugation at 15,000 rpm (21,130 g) at 4°C for 15 mins and the supernatant was incubated with pre-washed anti-HA magnetic beads (Thermo Scientific, 88837) on a rotor at 4°C overnight. Beads were washed five times with Triton X-100 lysis buffer and eluted in 2x SDS sample buffer (0.25 M Tris-HCl pH6.8, 10% SDS, 50% glycerol, 0.02% bromophenol blue) supplemented with 100 mM DTT and incubated at 65°C for 10 mins before western blot analysis.

Concanavalin A enrichment

Cleared cell lysates (i.e., supernatant) were incubated with 20 μl concanavalin A sepharose (Sigma-Aldrich, C9017-25ML) on a rotor at 4°C overnight. Beads were pelleted at 4000 rpm (1500 g) for 2 min at 4°C and washed five times with Triton X-100 lysis buffer. Glycoproteins were eluted with 2x LDS buffer (Invitrogen) supplemented with 25% sucrose and 50 mM DTT and samples were incubated at 65°C for 10 mins before western blot analysis using 4-12% Bis-Tris NuPAGE gradient gels (Invitrogen).

SDS-PAGE and western blotting

MOPS running buffer (50 mM MOPS, 50 mM Tris, 0.1 % SDS, 1 mM EDTA) was used for Bis-Tris gels, while Tris-Glycine running buffer (25 mM Tris,192 mM glycine, 0.1% SDS) was used for Novex 8-16% Tris-Glycine Mini Gels with WedgeWell format (Thermo Scientific). Proteins were then transferred to a methanol activated polyvinylidene difluoride (PVDF) membrane (Millipore) in Bis-Tris or Tris-Glycine transfer buffers. 5% milk in PBST (0.1% Tween 20) was used for blocking and antibody incubation. Membranes were incubated with secondary antibodies at the room temperature for 1 hr and washed with PBST. Blots were quantified using ImageJ.

The following antibodies were used: anti-ADAM17, rabbit polyclonal (Abcam, ab39162); anti-RFP (to detect mScarlet), mouse monoclonal (Chromotek, 6g6); anti-beta-actin, mouse monoclonal (Santa Cruz, sc-47778); anti-HA-HRP, rat monoclonal (clone 3F10) (Roche, 12013819001); anti-V5-Tag (D3H8Q), rabbit monoclonal (CST, 13202S); anti-beta-actin-HRP, mouse monoclonal (Sigma-Aldrich, A3854); anti-rabbit-HRP, goat polyclonal (CST, 7074); anti-mouse-HRP, horse polyclonal (CST, 7076).

Alkaline phosphatase—Shedding assay

iRhom1/iRhom2 double knockout (DKO) HEK 293T cells were seeded in poly-(L)-lysine (PLL, Sigma-Aldrich) coated 24-well plates in triplicates 24 hours before transfection. 150 ng alkaline phosphatase (AP)-conjugated substrates were co-transfected with 200 ng iRhom2 constructs using FuGENE® HD (Promega, E2312). 24 hours after transfection, cells were washed twice with PBS and incubated overnight in 300 μl phenol red-free OptiMEM (Gibco, 11058-021) supplemented with 2 μM GW280264X (GW) (Generon, AOB3632-5) or GI254023X (GI) (Sigma, SML0789-5MG) when indicated. The supernatants were then collected, and cells were lysed in 300 μl Triton X-100 lysis buffer supplemented with EDTA-free protease inhibitor mix (Roche) and 10 mM 1,10-phenanthroline (Sigma-Aldrich, 131377–5G). 100 μl supernatant and 100 μl diluted cell lysates were independently incubated with 100 μl AP substrate p-nitrophenyl phosphate (PNPP) (Thermo Scientific, 37620) at room temperature and the absorbance was measured at 405 nm by a plate reader (SpectraMax M3, Molecular Devices). The percentage of substrate release was calculated by dividing the signal from the supernatant by the total signal (supernatant and cell lysate).

Peptide substrate cleavage assay

The protease activity of the purified ADAM17 complex was measured using a fluorogenic peptide substrate Mca-PLAQAV-Dpa-RSSSR-NH2 (R&D Systems, catalog #ES003) (Figures S5A and S5B). The reaction was carried out in buffer containing 30 mM Tris pH 8, 0.05 % digitonin, and 3 μM $ZnCl_2$. Purified complexes were diluted to 0.24 μM in reaction buffer. When indicated, TAPI-0 (R&D Systems, catalog #5523) was supplemented to 10 μM to assess its inhibition. Different concentrations of fluorogenic peptide were added to initiate the reaction, and fluorescence signal was recorded continuously for 60 mins at 30 °C by a Synergy H1 microplate reader (BioTek) with excitation wavelength of 320 nm and emission wavelength of 405 nm. To quantify the peptide cleavage rate, mean values and, standard deviation from three independent measurements were calculated. Data and kinetic parameters were analyzed in GraphPad Prism 8.

QUANTIFICATION AND STATISTICAL ANALYSIS

In AP-shedding assays, error bars represent standard deviations (n=3, three transfectants). For quantification of western blots, error bars represent standard deviations (n=3, three independent experiments). Unless otherwise mentioned, a Dunnett's test is performed by computing a Student's t-statistic for each experimental, or treatment, group to compare each of a number of treatments with a single control, as described in each figure legend. ****=p<0.0001, **=p<0.01, *=p<0.05, ns= not significant. Western blots were quantified using ImageJ. Statistical analyses of data were performed using GraphPad Prism 10. Detailed quantification methods and statistical analyses performed are described in the figure legends.

Molecular Cell, Volume *84*

Supplemental information

Cryo-EM reveals that iRhom2 restrains

ADAM17 protease activity to control the release

# of growth factor and inflammatory signals

Fangfang Lu, Hongtu Zhao, Yaxin Dai, Yingdi Wang, Chia-Hsueh Lee, and Matthew Freeman

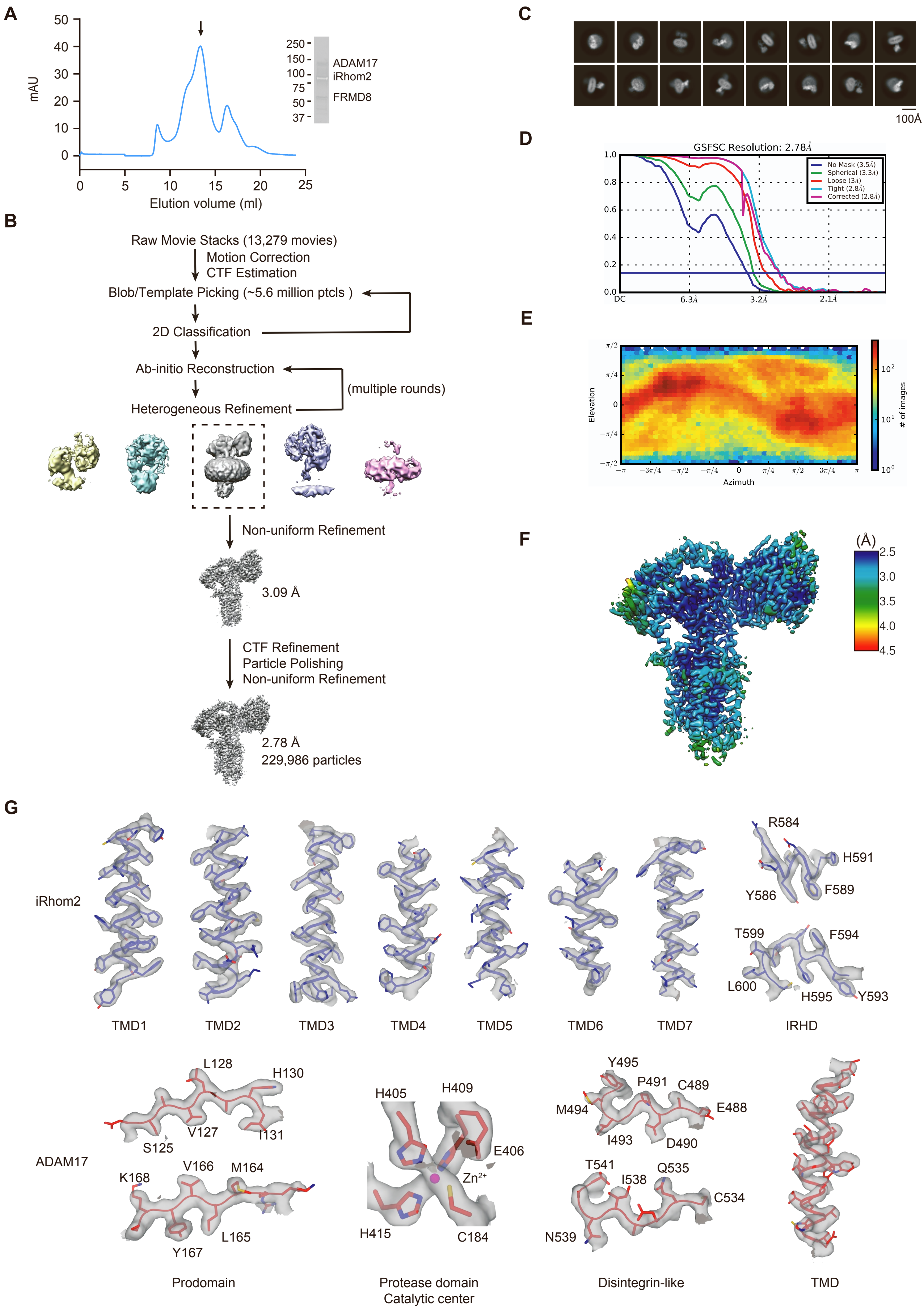
A
mAU
50
40
30
20
10
0
0
5
10
15
20
25
Elution volume (ml)
250
150
100
75
50
37
ADAM17
iRhom2
FRMD8
B
Raw Movie Stacks (13,279 movies)
Motion Correction
CTF Estimation
Blob/Template Picking (~5.6 million ptcls)
2D Classification
Ab-initio Reconstruction
(multiple rounds)
Heterogeneous Refinement
Non-uniform Refinement
3.09 Å
CTF Refinement
Particle Polishing
Non-uniform Refinement
2.78 Å
229,986 particles
C
100Å
D
GSFSC Resolution: 2.78Å
No Mask (3.5Å)
Spherical (3.3Å)
Loose (3Å)
Tight (2.8Å)
Corrected (2.8Å)
DC
6.3Å
3.2Å
2.1Å
E
Elevation
Azimuth
# of images
F
(Å)
2.5
3.0
3.5
4.0
4.5
G
iRhom2
TMD1
TMD2
TMD3
TMD4
TMD5
TMD6
TMD7
R584
H591
Y586
F589
T599
F594
L600
H595
Y593
IRHD
ADAM17
L128
H130
V127
I131
S125
K168
V166
M164
L165
Y167
Prodomain
H405
H409
E406
Zn2+
H415
C184
Protease domain
Catalytic center
Y495
P491
C489
M494
E488
I493
D490
T541
I538
Q535
C534
N539
Disintegrin-like
TMD

Figure S1 Cryo-EM analyses of the human ADAM17/iRhom2 sheddase complex, related to Figure 1.

(A) Representative size exclusion chromatography profile and SDS-PAGE analysis of the purified ADAM17/iRhom2 complex. **(B)** Summary of image processing procedures of the ADAM17/iRhom2 complex. All procedures were done in cryoSPARC, except for particle polishing which was done in RELION. **(C)** Representative 2D class averages. **(D)** Fourier shell correlation (FSC) curves between two half maps. **(E)** Angular distribution of particles for the final 3D reconstructions. **(F)** Local resolution of the cryo-EM density. The map is colored according to local resolution, estimated using cryoSPARC. **(G)** cryo-EM densities of iRhom2 and ADAM17.

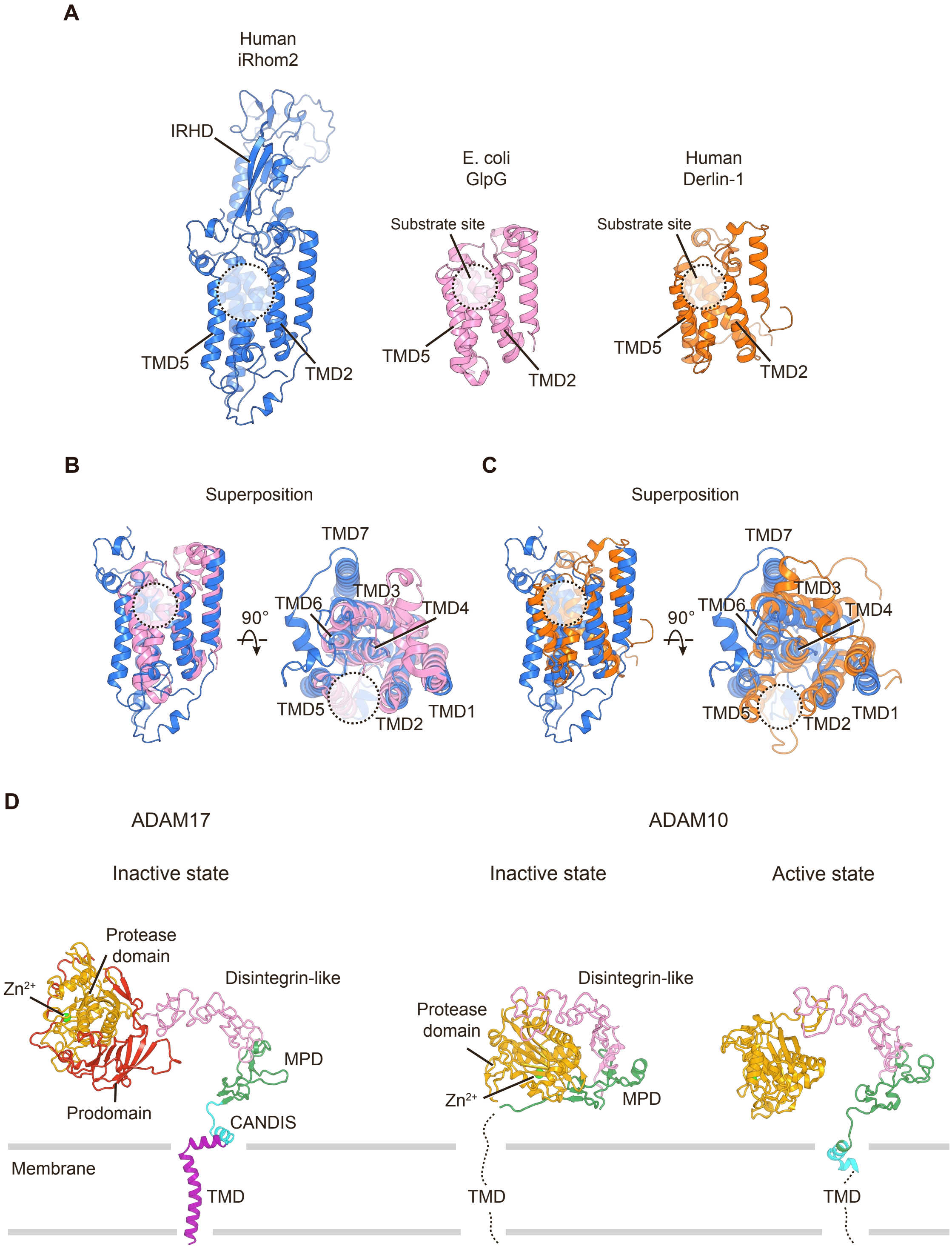
A
Human
iRhom2
IRHD
TMD5
TMD2
E. coli
GlpG
Substrate site
TMD5
TMD2
Human
Derlin-1
Substrate site
TMD5
TMD2
B
Superposition
TMD7
TMD3
TMD6
TMD4
90°
TMD5
TMD2
TMD1
C
Superposition
TMD7
TMD3
TMD6
TMD4
90°
TMD5
TMD2
TMD1
D
ADAM17
Inactive state
Protease
domain
Zn2+
Disintegrin-like
MPD
Prodomain
CANDIS
Membrane
TMD
ADAM10
Inactive state
Disintegrin-like
Protease
domain
Zn2+
MPD
TMD
Active state
TMD

Figure S2 Comparison of ADAM17/iRhom2 complex with related structures, related to Figures 1 and 2.

(A) Structures of human iRhom2, *E. coli* GlpG (PDB: 2IC8) and human derlin-1 (PDB: 7CZB). **(B)** Superposition between iRhom2 and GlpG. **(C)** Superposition between iRhom2 and derlin-1. **(D)** Structural comparison of ADAM17 and ADAM10 (PDB: 6BE6 and 8ESV). The three structures are aligned based on disintegrin-like domains. Note that ADAM10's protease domain interacts with the MPD in the inactive state. Such an interaction does not exist in the ADAM17 sheddase complex. The catalytic Zn^{2+} ions are shown as green spheres. The approximate membrane boundaries are represented by grey bars.

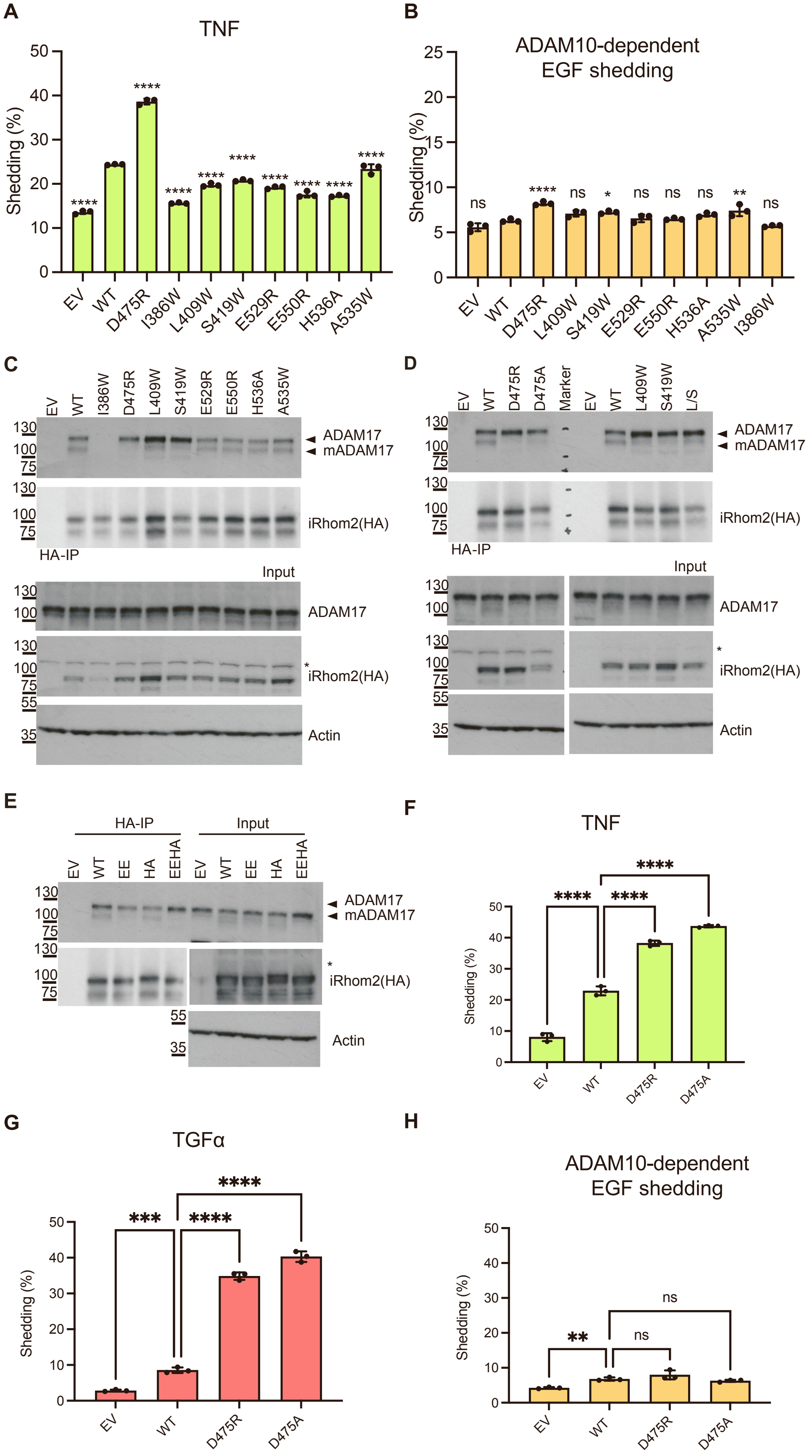
A
TNF
Shedding (%)
0
10
20
30
40
50
****
EV
WT
D475R
I386W
L409W
S419W
E529R
E550R
H536A
A535W
B
ADAM10-dependent
EGF shedding
Shedding (%)
0
5
10
15
20
25
ns
*
**
EV
WT
D475R
L409W
S419W
E529R
E550R
H536A
A535W
I386W
C
EV
WT
I386W
D475R
L409W
S419W
E529R
E550R
H536A
A535W
130
100
75
55
35
ADAM17
mADAM17
iRhom2(HA)
HA-IP
Input
ADAM17
Actin
D
EV
WT
D475R
D475A
Marker
L409W
S419W
L/S
E
HA-IP
Input
EE
HA
EEHA
F
TNF
G
TGFα
***
H
ADAM10-dependent
EGF shedding
D475A

Figure S3 Mutations at D475 in iRhom2 increases ADAM17-dependent growth factor and cytokine release, without affecting ADAM10 activity, related to Figures 3 and 4.

(A, B, F-H) iRhom1/2 DKO HEK cells were transfected with empty vector (EV) or different iRhom2 single point mutants together with AP-tagged ADAM17 substrates, tumor necrosis factor (TNF), transforming growth factor alpha (TGFα), or ADAM10 substrate epidermal growth factor (EGF). The growth medium was collected overnight and used for the AP-shedding assay. Error bars represent standard deviations (n=3, three transfectants). A Dunnett's test is performed by computing a Student's t-statistic for each transfection condition compared to the WT iRhom2 condition. ****=p<0.0001, ***=p<0.001, **=p<0.01, *=p<0.05, ns=not significant. Data are representative of three independent experiments. **(C-E)** iRhom1/2 DKO HEK cells were transfected with empty vector (EV) or different iRhom2 variants (L/S: L409W+ S419W, EE: E529R/E550R, HA: H536A/A535W, EEHA: E529R/E550R/H536A/A535W). HA-based immunoprecipitates and lysates were blotted for ADAM17, HA (iRhom2) and Actin. * indicates non-specific bands.

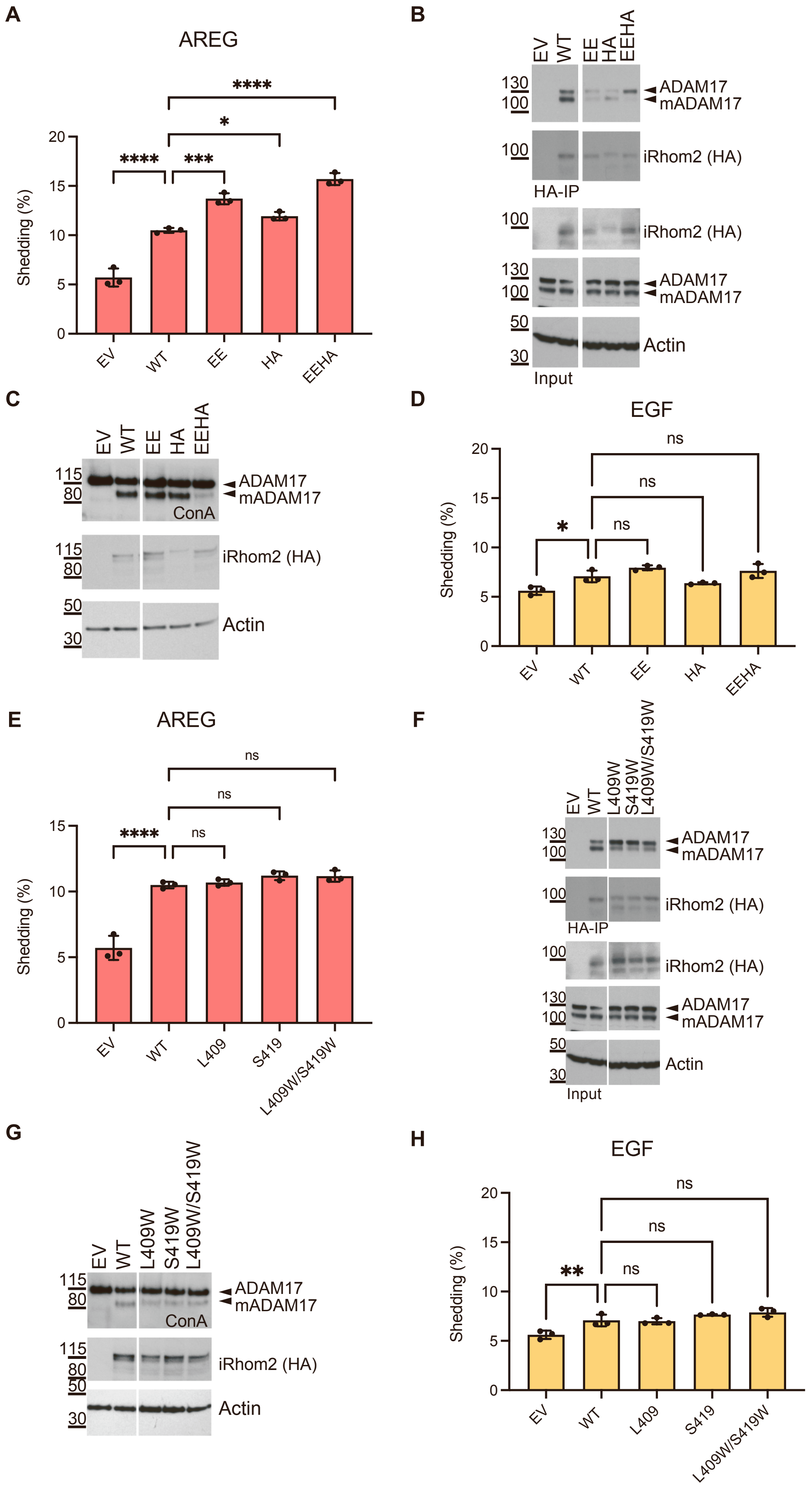
A
AREG
Shedding (%)
EV
WT
EE
HA
EEHA
****
*
***
B
HA-IP
Input
ADAM17
mADAM17
iRhom2 (HA)
Actin
C
ConA
115
80
50
30
D
EGF
ns
E
L409
S419
L409W/S419W
F
L409W
S419W
G
H
**

Figure S4 Disrupting interaction at the Interfaces 2 and 4 had limited effects on ADAM17 activity, related to Figure 3.

(A) and **(D),** iRhom1/2 DKO HEK cells were transfected with empty vector (EV) or different iRhom2 Interface 2 mutants (EE: E529R/E550R, HA: H536A/A535W, EEHA: E529R/E550R/H536A/A535W) together with AP-tagged AREG as ADAM17 substrate or AP-tagged EGF as ADAM10 substrate in (D). Error bars represent standard deviations (n=3，three transfectants). **(B)** iRhom1/2 DKO HEK cells were transfected with WT or iRhom2 Interface 2 mutants (EE, HA, EEHA). HA-based immunoprecipitates and lysates were blotted with endogenous ADAM17, HA (iRhom2), and actin. **(C)** Concanavalin A (ConA) enrichment was performed to the lysates from the shedding assay in (A). **(E)** and **(H),** iRhom1/2 DKO HEK cells were transfected with empty vector (EV) or different iRhom2 Interface 4 mutants (L409W, S419W, L/S: L409W+ S419W) together with AP-tagged AREG as ADAM17 substrate or AP-tagged EGF as ADAM10 substrate in (H). Error bars represent standard deviations (n=3, three transfectants). **(F)** iRhom1/2 DKO HEK cells were transfected with WT or iRhom2 Interface 4 mutants (L409W, S419W, L/S). HA-based immunoprecipitates and lysates were blotted with endogenous ADAM17, HA (iRhom2), and actin. **(G)** Concanavalin A (ConA) enrichment was performed to the lysates from the shedding assay in (E). Data are representative of three independent experiments (A-H). For figures A, D, E, H, a Dunnett's test is performed by computing a Student's t-statistic for each transfection condition compared to the WT iRhom2 condition. ****=p<0.0001, ***=p<0.001, **=p<0.01, *=p<0.05, ns= not significant. Note: EV, WT conditions are on the same immunoblot as the different mutant conditions, with superfluous lanes removed to make comparison easier. (B) and (F) show different mutants from one HA-IP experiment and therefore share the same control conditions.

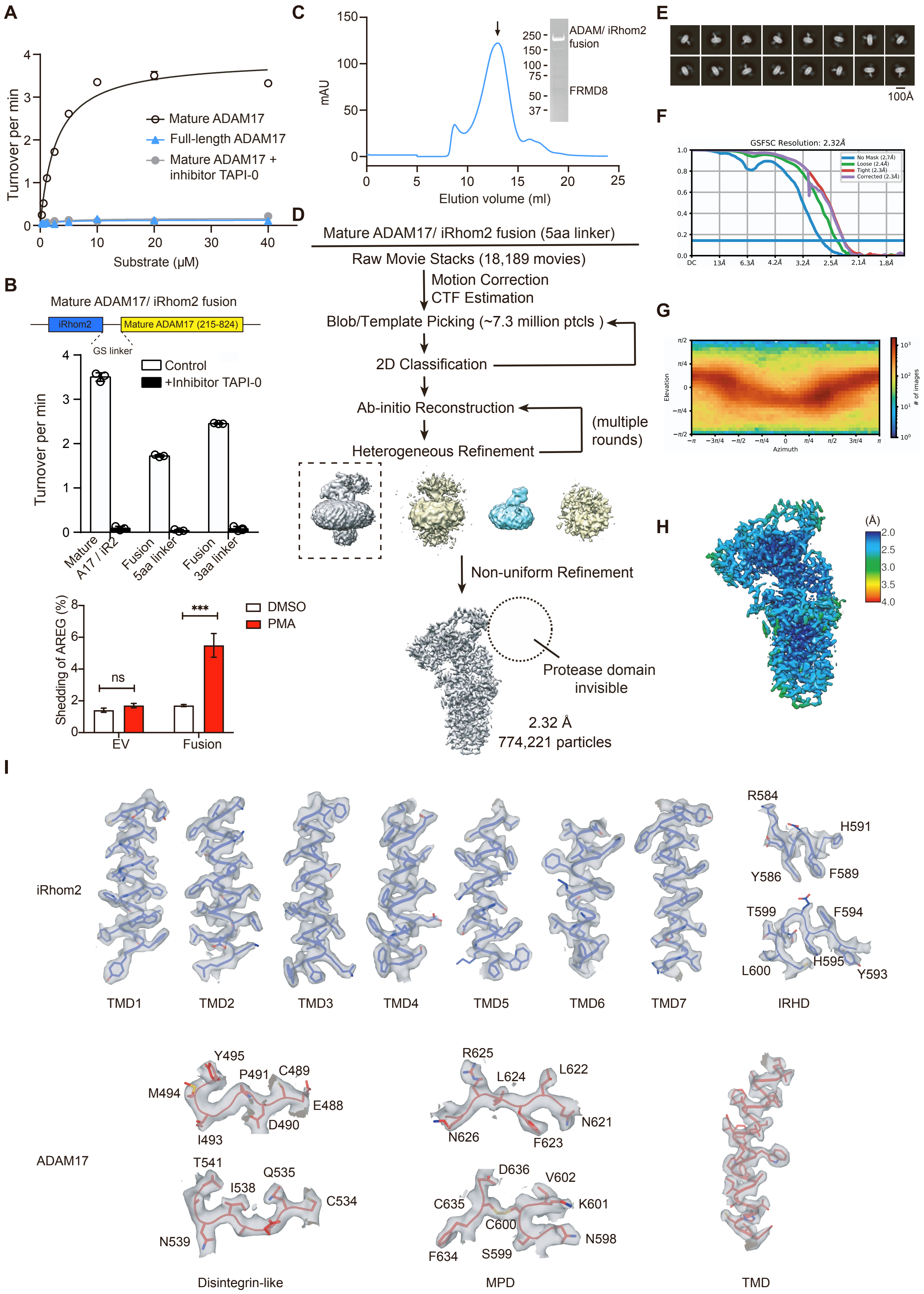
A
Turnover per min
Substrate (μM)
Mature ADAM17
Full-length ADAM17
Mature ADAM17 +
inhibitor TAPI-0
B
Mature ADAM17/ iRhom2 fusion
iRhom2
Mature ADAM17 (215-824)
GS linker
Control
+Inhibitor TAPI-0
Turnover per min
Mature A17 / iR2
Fusion 5aa linker
Fusion 3aa linker
Shedding of AREG (%)
DMSO
PMA
ns
***
EV
Fusion
C
mAU
Elution volume (ml)
ADAM/ iRhom2
fusion
FRMD8
D
Mature ADAM17/ iRhom2 fusion (5aa linker)
Raw Movie Stacks (18,189 movies)
Motion Correction
CTF Estimation
Blob/Template Picking (~7.3 million ptcls)
2D Classification
Ab-initio Reconstruction
Heterogeneous Refinement
(multiple rounds)
Non-uniform Refinement
Protease domain invisible
2.32 Å
774,221 particles
E
100Å
F
GSFSC Resolution: 2.32Å
No Mask (2.7Å)
Loose (2.4Å)
Tight (2.3Å)
Corrected (2.3Å)
G
Elevation
Azimuth
# of images
H
(Å)
2.0
2.5
3.0
3.5
4.0
I
iRhom2
TMD1
TMD2
TMD3
TMD4
TMD5
TMD6
TMD7
R584
H591
Y586
F589
T599
F594
L600
H595
Y593
IRHD
ADAM17
Y495
P491
C489
M494
E488
D490
I493
T541
Q535
I538
C534
N539
Disintegrin-like
R625
L624
L622
N626
N621
F623
D636
V602
C635
K601
C600
N598
F634
S599
MPD
TMD

Figure S5 Functional and cryo-EM analyses of mature ADAM17/iRhom2 complexes, related to Figure 5.

(A) Protease activity of the purified mature ADAM17/iRhom2 complex and the full length. **(B)** Upper panel: Protease activity of the purified, mature ADAM17/iRhom2 fusions. Lower panel: AP-shedding assay of iRhom1/2 DKO HEK cells which were transfected with empty vector (EV) or an iRhom2-matureADAM17 fusion construct (Fusion) together with the ADAM17 substrate alkaline phosphatase (AP)-tagged AREG. A Student's t-statistic test was performed. ***=$p<0.001$, ns= not significant; $n=3$, three transfectants. In (A) and (B), error bars represent standard deviations. **(C)** Representative size exclusion chromatography profile and SDS-PAGE analysis of the mature ADAM17/iRhom2 fusion (5 aa linker construct). **(D)** Summary of image processing procedures of the mature ADAM17/iRhom2 fusion (5 aa linker construct). All procedures were done in cryoSPARC, except for particle polishing which was done in RELION. **(E)** Representative 2D class averages. **(F)** Fourier shell correlation (FSC) curves between two half maps. **(G)** Angular distribution of particles for the final 3D reconstructions. **(H)** Local resolution of the cryo-EM density. The map is colored according to local resolution, estimated using cryoSPARC. **(I)** cryo-EM densities of iRhom2 and mature ADAM17.

Table S1. Table of mutants, related to Figure 3.

Interface	iRhom2 domain	iRhom2 isoform2	Potential ADAM17 interacting residues	ADAM17 domain
4	IRHD	L409W<br>S419W	V120<br>H118	Prodomain
3	IRHD	D475R	S63 or T64 or T62	Prodomain
2	IRHD	E529R<br>E550R<br>H536A<br>A535W	K626<br>R625<br>K628<br>S590	Membrane proximal domain (MPD)
1	TMD1	I386W	V673, V676, L677	Transmembrane domain (TMD)
Rationale of mutagenesis design	To ensure disruption of the interaction interface, the changes of amino acids were designed to be profound. All small residues (L, S, A, I, V) were mutated to a bulky residue W. For charged residues, the charge was reversed. For instance, an E was mutated into an R or vice versa. In addition, H was mutated to A.			
